# Cardiovascular health and four epigenetic clocks

**DOI:** 10.1186/s13148-022-01295-7

**Published:** 2022-06-09

**Authors:** Yun-Hsiang Lo, Wan-Yu Lin

**Affiliations:** 1 Institute of Epidemiology and Preventive Medicine, College of Public Health, National Taiwan University, Room 501, No. 17, Xu-Zhou Road, Taipei, 100 Taiwan; 2 Master of Public Health Degree Program, College of Public Health, National Taiwan University, Taipei, Taiwan; 3 Department of Public Health, College of Public Health, National Taiwan University, Taipei, Taiwan

**Keywords:** Aging, Chronological age, DNA methylation, Methylation clock

## Abstract

**Background:**

Cardiovascular health (CVH) was defined by the American Heart Association as an integrative idealness of seven clinical or lifestyle factors. Based on populations of European ancestry, recent studies have shown that ideal CVH is associated with a slower aging rate. The aging rate is measured by levels of epigenetic age acceleration (EAA), usually obtained from the residuals of regressing DNA methylation (DNAm) age on chronological age. However, little has been known about the association of CVH with biological aging in Asian populations.

**Methods and results:**

We here analyzed blood DNAm data and clinical/lifestyle factors of 2474 Taiwan Biobank (TWB) participants, to investigate the association of CVH with EAA. CVH was assessed by seven components: smoking status, physical activity, dietary habits, body mass index, total cholesterol, fasting glucose, and blood pressure levels. Four measures of EAA were applied, among which two were based on the first-generation DNAm clocks (HannumEAA and IEAA) and two were based on the second-generation clocks (PhenoEAA and GrimEAA). After excluding 276 individuals with cardiovascular diseases, we regressed EAA on the CVH score (ranging from 0 to 7, integrating the abovementioned seven components) while adjusting for sex, drinking status, and educational attainment.

Our results showed that a decrease in one point in the CVH score was associated with a 0.350-year PhenoEAA (*p* = 4.5E−4) and a 0.499-year GrimEAA (*p* = 4.2E−15). By contrast, HannumEAA and IEAA were not significantly associated with the CVH score. We have obtained consistent results within each generation of epigenetic clocks.

**Conclusions:**

This is one of the first studies to comprehensively investigate the associations of CVH with four epigenetic clocks. Our TWB data showed that ideal CVH is associated with lower levels of EAA calculated according to the second-generation epigenetic clocks (PhenoEAA and GrimEAA). Having an ideal CVH status can lower EAA and reduce the risk of aging-related disorders.

**Supplementary Information:**

The online version contains supplementary material available at 10.1186/s13148-022-01295-7.

## Introduction

Cardiovascular health (CVH) was defined by the American Heart Association (AHA) as an integrative idealness of four lifestyle factors (cigarette smoking, physical activity, body mass index (BMI), and dietary habits) and three clinical factors (fasting glucose, blood pressure, and total cholesterol level) [1]. Ideal CVH is associated with lower risks of cardiovascular diseases (CVDs) and chronic diseases and decreased all-cause mortality [1, 2]. Ideal CVH is also associated with more extended longevity and a better quality of life [3, 4].

Epigenetic change may reflect the idealness of CVH [5, 6]. One of the epigenetic mechanisms is DNA methylation (DNAm), which can dynamically express human aging-related physiological changes [7–9]. Therefore, DNAm has been used to estimate human biological age [9–13]. Epigenetic age (i.e., the so-called DNAm age/clock) measures biological age to predict people’s healthspan and lifespan. For example, Hannum et al. constructed a DNAm age estimator (i.e., the so-called “Hannum’s clock”) from whole-blood samples by selecting 71 cytosine-phosphate-guanine (CpG) sites that are highly predictive of chronological age [10]. Horvath further developed a multi-tissue DNAm clock with an average of 96% correlation between the DNAm age and chronological age [11]. Horvath used the elastic-net regularized regression [14] to select 353 CpGs by regressing a transformed version of chronological age on 21,369 CpG sites. These CpGs were therefore used to form a DNAm age (i.e., the so-called “Horvath’s clock”) [11]. Horvath showed that an average of 36 years of age acceleration was observed in 20 different cancer types [11].

Hannum’s clock and Horvath’s clock are regarded as the first-generation DNAm clocks because they are predictive of chronological age [15, 16]. The second-generation DNAm clocks, on the other hand, aim to estimate biological age that can better reflect disease morbidity and mortality [15–17]. For example, Levine et al. used a two-step approach to develop the “PhenoAge” [12]. They first selected 9 biomarkers and chronological age by regressing the hazard of aging-related mortality on 43 candidate markers (including 42 clinical biomarkers and chronological age) through a regularized Cox regression model. These 9 biomarkers and chronological age were then aggregated into a “phenotypic age,” reflecting one’s physiological conditions better than chronological age [12, 16]. In the second step, Levine et al*.* selected 513 CpGs from 20,169 CpGs as predictors of the phenotypic age. A weighted average of these 513 CpGs formed a DNAm clock (i.e., the so-called “PhenoAge”) [12].

Another second-generation DNAm clock is “GrimAge” [13]. A total of 7 DNAm-based surrogate markers of plasma proteins and smoking pack-years were found to be associated with time-to-death. These eight sets of surrogate markers were based on 1,030 unique CpGs. GrimAge was developed by aggregating the information of these 1,030 CpGs. Therefore, GrimAge is highly predictive of disease mortality and can reflect more aging-related physiological conditions [17].

Epigenetic age acceleration (EAA) is a novel measure to evaluate human aging rate, usually quantified by the residuals of regressing DNAm age on chronological age [18–21]. In this way, the EAA calculation will be robust to different measurement platforms and normalization methods [18–21]. A positive EAA implies that an individual is aging faster than the expected aging rate regarding his/her chronological age. Recent studies have shown that ideal CVH is associated with lower EAA in European ancestry populations [5, 6]. However, little has been known about the association of CVH with biological aging in Asian people.

Here, we investigated the association of ideal CVH with four measures of EAA based on the DNAm data from 2474 Taiwan Biobank (TWB) participants. Two measures of EAA were calculated according to the first-generation DNAm clocks, among which HannumEAA was estimated from Hannum’s clock [11] and IEAA was obtained from Horvath’s clock [10]. The other two measures of EAA were calculated according to the second-generation DNAm clocks, among which PhenoEAA was gauged from PhenoAge [12] and GrimEAA was calculated from GrimAge [13]. We obtained four measures of EAA by uploading the DNAm data to the online DNAm Age Calculator created by Horvath’s laboratory (https://dnamage.genetics.ucla.edu/new). Similar to previous studies [5, 6], after excluding participants with extreme EAA levels and participants diagnosed with CVDs or leukemia, we regressed EAA (the response variable) on the CVH score (the explanatory variable) [1] while adjusting for sex, drinking status, and educational attainment. We aimed to test the associations of the CVH scores with EAA among Asian individuals without CVDs.

Both the 7-point and 14-point scales of the CVH score were applied according to the definition from the AHA [1]. However, the scoring criteria of BMI and ideal diet behaviors were adjusted to accommodate the standards of Asians (will be described in “Methods” section). Because only ~ 60% of the 2474 TWB participants provided their dietary habits, we also calculated 6-point and 12-point CVH scores by excluding the component of “ideal diet” (will be described in “Methods” section).

## Results

Table 1 presents the basic characteristics of the 2,474 TWB participants stratified by tertiles of GrimEAA. The results stratified by tertiles of PhenoEAA, IEAA, and HannumEAA were shown in Additional file 1: Tables S1–S3, respectively. Participants were aged from 30 to 70 years. All seven components of the CVH score demonstrated significantly different distributions across the three GrimEAA tertile groups (*p* < 0.05, Table 1).

**Table 1:** Basic characteristics of the 2474 TWB participants stratified by tertiles of GrimEAA

	Overall	GrimEAA T1 (< − 1.64 years)^Ref^	GrimEAA T2 (− 1.64 to 0.88 years)	GrimEAA T3 (> 0.88 years)
*N* (male %)	2474 (50.24%)	825 (27.39%)	824 (48.18%)***	825 (75.15%)***
Chronological age (standard deviation, SD)	49.76 (11.08)	50.52 (10.98)	48.63 (11.02)***	50.12 (11.16)
*Education (%)*				
Illiterate	5 (0.20%)	2 (0.24%)	3 (0.36%)	0 (0.00%)
No formal education but literate	2 (0.08%)	1 (0.12%)	1 (0.12%)	0 (0.00%)
Primary school graduate	95 (3.84%)	33 (4.00%)	27 (3.28%)	35 (4.24%)
Junior high school graduate	137 (5.54%)	49 (5.94%)	43 (5.22%)	45 (5.45%)
Senior high school graduate	718 (29.02%)	242 (29.33%)	223 (27.06%)	253 (30.67%)
College graduate	1254 (50.69%)	413 (50.06%)	433 (52.55%)	408 (49.45%)
Master’s or higher degree	261 (10.55%)	84 (10.18%)	94 (11.41%)	83 (10.06%)
*7 components of the CVH score (%)*				
Smoking status—never	1614 (65.24%)	693 (84.00%)	598 (72.48%)***	323 (39.15%)***
Smoking status—former^a^	312 (12.61%)	47 (5.70%)	89 (10.79%)***	176 (21.33%)***
Smoking status—current	283 (11.44%)	16 (1.94%)	29 (3.52%)	238 (28.85%)***
Ideal BMI^b^	1240 (50.12%)	508 (61.58%)	420 (50.91%)***	312 (37.82%)***
Ideal physical activity^c^	1092 (44.14%)	389 (47.15%)	347 (42.06%)*	356 (43.15%)
Ideal cholesterol level^d^	1444 (58.37%)	441 (53.45%)	503 (60.97%)**	500 (60.61%)**
Ideal fasting glucose level^e^	1951 (78.86%)	713 (86.42%)	667 (80.85%)**	571 (69.21%)***
Ideal blood pressure^f^	1315 (53.15%)	490 (59.39%)	447 (54.18%)*	378 (45.82%)***
Ideal diet^g^	456 (31.78% out of 1435)	185 (39.03% out of 474)	162 (32.40% out of 500)*	109 (23.64% out of 461)***
*6-point CVH score (%)*				
*N*	127 (5.13%)	824	824	823
0–1	329 (13.30%)	15 (1.82%)	24 (2.91%)	88 (10.67%)***
2	554 (22.39%)	76 (9.21%)	105 (12.73%)*	148 (17.94%)***
3	716 (28.94%)	151 (18.30%)	172 (20.85%)	231 (28.00%)***
4	586 (23.69%)	256 (31.03%)	257 (31.15%)	203 (24.61%)**
5	159 (6.43%)	258 (31.27%)	206 (24.97%)**	122 (14.79%)***
6	127 (5.13%)	68 (8.25%)	60 (7.28%)	31 (3.77%)***

^Ref^T1 (tertile 1, the reference group); T2 (or T3) compared with T1 based on the two-sample proportion test

^a^A former smoker was defined as an individual who has quitted smoking for at least 6 months

^b^Ideal BMI: body mass index less than 24 kg/m^2^, according to the criterion proposed by the Ministry of Health and Welfare, Taiwan

^c^Ideal physical activity was defined as performing 30 min of exercise (including leisure-time activities such as swimming, cycling, jogging, weight training, dancing, mountain climbing, etc.) at least 3 times a week

^d^Ideal cholesterol level was defined as a total cholesterol level less than 200 mg/dL

^e^Ideal fasting glucose level was defined as a fasting glucose level less than 100 mg/dL

^f^Ideal blood pressure was defined as a systolic blood pressure less than 120 mmHg and diastolic blood pressure below 80 mmHg

^g^Ideal diet was assessed according to the consumption of food categories, sodium, and fat intake

^*^p < 0.05; **p < 0.01; ***p < 0.001

The percentage of “ideal BMI” was larger in the lowest tertile (T1) than in the second (T2) or the highest tertile (T3) of GrimEAA (*p* < 0.001, Table 1). Similar results were also observed in PhenoEAA (*p* < 0.001, Additional file 1: Table S1), IEAA (*p* < 0.01 between T1 and T3, Additional file 1: Table S2), and HannumEAA (*p* < 0.01 between T1 and T2, *p* < 0.001 between T1 and T3, Additional file 1: Table S3). These results showed that an increased BMI value is associated with a faster biological aging rate, consistent with previous studies indicating that an increased BMI is associated with a shortened telomere length [22–24]. Telomere length is another well-known biological aging biomarker. A shortened telomere length can reflect an acceleration of the aging pace [25, 26].

Regarding the CVH component of “smoking status”, fewer never-smoking individuals were in the highest tertile than in the lowest tertile of GrimEAA (*p* < 0.001, Table 1), while more current or former smokers in the highest tertile than in the lowest tertile of GrimEAA (*p* < 0.001, Table 1). Similar results were also observed in the other three measures of EAA (Additional file 1: Tables S1–S3).

For the ideal fasting glucose level, more individuals were in the lowest tertile than in both the second (*p* < 0.01) and the highest tertile of GrimEAA (*p* < 0.001, Table 1). Significant differences between the lowest tertile and the second or highest tertile of GrimEAA were also observed in ideal blood pressure and ideal diet (*p* < 0.001, Table 1).

After excluding participants with CVDs (*n* = 276) or with extreme EAA values (*n* = 7 for HannumEAA; *n* = 1 for IEAA; *n* = 2 for PhenoEAA; *n* = 5 for GrimEAA; Methods), we performed multiple linear regression analysis to investigate the association between the CVH score and the four measures of EAA (HannumEAA, IEAA, PhenoEAA, and GrimEAA), respectively. Because our study sample contained no leukemia cases, no participants were excluded due to leukemia.

Table 2 shows the results of regressing the four measures of EAA on four different definitions of the CVH score while adjusting for sex, drinking status, and educational attainment (coefficients of covariates are presented in Additional file 1: Table S5). All models consistently demonstrated inverse associations between the CVH scores and the levels of EAA, as we can see from the negative regression coefficients of the CVH scores (Beta in Table 2). However, only EAA derived from the second-generation DNAm clocks (i.e., PhenoEAA and GrimEAA) led to significant inverse associations with all four definitions of the CVH score (*p* ≤ 4.5E−4).

**Table 2:** Results of regressing four measures of EAA on the CVH score

	1st generation of epigenetic clocks						2nd generation of epigenetic clocks					
	IEAA^a^			HannumEAA^a^			PhenoEAA^a^			GrimEAA^a^		
	Beta	95% CI	*p*	Beta	95% CI	*p*	Beta	95% CI	*p*	Beta	95% CI	*p*
*Seven-component CVH scores*^b^												
CVH score (2-level, 7-point)	− 0.101	[− 0.2475, 0.0458]	0.177	− 0.122	[− 0.2709, 0.0269]	0.108	− 0.350	[− 0.5459, − 0.1550]	4.5E−4	− 0.499	[− 0.6222, − 0.3758]	4.2E−15
CVH score (3-level, 14-point)	− 0.074	[− 0.1670, 0.0194]	0.120	− 0.083	[− 0.1773, 0.0119]	0.087	− 0.268	[− 0.3919, − 0.1439]	2.4E−5	− 0.364	[− 0.4419, − 0.2865]	1.5E−19
*Six-component CVH scores*^c^												
CVH score (2-level, 6-point)	− 0.086	[− 0.2093, 0.0366]	0.169	− 0.088	[− 0.2092, 0.0324]	0.151	− 0.388	[− 0.5528, − 0.2238]	3.9E−6	− 0.526	[− 0.6289, − 0.4222]	6.1E−23
CVH score (3-level, 12-point)	− 0.068	[− 0.1440, 0.0085]	0.082	− 0.065	[− 0.1396, 0.0102]	0.091	− 0.278	[− 0.3798, − 0.1761]	9.6E−8	− 0.377	[− 0.4407, − 0.3136]	2.1E−30

^a^IEAA (i.e., intrinsic EAA), HannumEAA, PhenoEAA, and GrimEAA were calculated according to the four epigenetic clocks: Horvath’s clock [11], Hannum et al.’s clock [10], Levine et al.’s PhenoAge [12], and Lu et al.’s GrimAge [13], respectively

^b^The seven-component CVH score was calculated according to the definition of CVH from the American Heart Association (AHA)

^c^Because 42% of the 2474 TWB participants were surveyed by the simplified questionnaire without diet information, the six-component CVH score was calculated without the “ideal diet score.” Other components followed the same definition of the CVH score from the AHA

For the 7-point CVH score, a decrease in one point in the CVH score was associated with a 0.350-year PhenoEAA (*p* = 4.5E−4, 95% CI: 0.1550–0.5459; Table 2) and a 0.499-year GrimEAA (*p* = 4.2E−15, 95% CI: 0.3758–0.6222; Table 2). For the 14-point CVH score, a decrease in one point in the CVH score was associated with a 0.268-year PhenoEAA (*p* = 2.4E−5, 95% CI: 0.1439–0.3919; Table 2) and a 0.364-year GrimEAA (*p* = 1.5E−19, 95% CI: 0.2865–0.4419; Table 2).

By contrast, IEAA (*p* = 0.177 for 7-point; *p* = 0.120 for 14-point) and HannumEAA (*p* = 0.108 for 7-point; *p* = 0.087 for 14-point) were not significantly associated with the CVH scores (Table 2). We obtained similar results even when the CVH score was defined by six components (i.e., excluding diet information largely missing in the TWB data), as shown in the bottom two rows of Table 2. For the 6-point CVH score, a decrease in one point in the CVH score was associated with a 0.388-year PhenoEAA (*p* = 3.9E−6, 95% CI: 0.2238–0.5528) and a 0.526-year GrimEAA (*p* = 6.1E−23, 95% CI: 0.4222–0.6289). For the 12-point CVH score, a decrease in one point in the CVH score was associated with a 0.278-year PhenoEAA (*p* = 9.6E−8, 95% CI: 0.1761–0.3798) and a 0.377-year GrimEAA (*p* = 2.1E−30, 95% CI: 0.3136–0.4407). Again, IEAA and HannumEAA were not significantly associated with the six-component CVH scores (Table 2).

## Discussion

In this study, we evaluated the associations of CVH with four measures of EAA through DNAm, clinical, and lifestyle data of 2,474 TWB participants. We found that, among the participants free of CVD, higher CVH scores were associated with lower EAA calculated based on PhenoEAA [12] and GrimEAA [13] (second-generation epigenetic age). In contrast, the associations were not significant between the CVH scores and EAA calculated based on Horvath’s [11] and Hannum’s [10] clocks (first-generation epigenetic age). This result was robust to various definitions of the CVH scores (7-point, 14-point, 6-point, and 12-point scales).

The lack of significance of the association when shifting the EAA measurement from the second-generation epigenetic clocks to the first-generation clocks may result from the different purposes of the two generations of clocks. The second-generation epigenetic clocks were trained to reflect aging-related physiological conditions, whereas the first-generation clocks aimed to predict human chronological age [16]. Increased chronological age is an important risk factor for many diseases and cancers. However, it does not account for the heterogeneity of physiological complexity among individuals of the same age. The EAA calculated based on the first-generation clocks would tend to “inherit” this “ignorance of physiological conditions.” By contrast, the second-generation clocks-based EAA is more sensitive to the impacts of CVH [13].

This is one of the first studies to investigate the association of CVH with EAA in Asian populations (specifically, the Taiwanese population). As most studies of DNAm age were focused on European ancestry populations [10–13, 15, 17, 27], our DNAm data from the 2,474 TWB participants were unique as far as the ethnicity is concerned.

Recently, Pottinger et al*.* [5] investigated the associations of the CVH score with the first-generation clocks-based EAA [10, 11] in 2,170 postmenopausal women (aged from 50 to 79 years) from the Women’s Health Initiative (WHI) cohort [28]. Unlike our results, Pottinger et al*.* [5] showed that ideal CVH was associated with the first-generation clocks-based EAA [10, 11]. The differences between the two data sets (WHI [28] and TWB) may result in these inconsistent results. First, among the 2,170 individuals analyzed by Pottinger et al*.* [5], 50% were non-Hispanic white, 25% were African Americans, and 15% were Hispanics. In contrast, most TWB individuals were of Han Chinese ancestry [29]. Second, the 2,170 individuals in Pottinger et al*.*’s study were all postmenopausal women aging from 50 to 79 years [5], whereas our data included 2,474 men (50.24%) or women (49.76%) aging from 30 to 70 years. The TWB data presented a broader spectrum of sex and age than the WHI cohort [28].

Joyce et al*.* also investigated the associations of the CVH score with GrimEAA at 3,224 participants from two cohort studies [6]. DNAm data were measured from 2,106 offspring participants of the Framingham Heart Study (FHS) at exam 8 (2005–2008) and 1,118 randomly-selected individuals of Coronary Artery Risk Development in Young Adults (CARDIA) study at Y15 (2000–2001) and Y20 (2005–2006), respectively. Their cross-sectional and longitudinal analyses both showed significant associations between the CVH score and GrimEAA (*p* < 0.01). Our results were in line with their study, indicating that the association of CVH with GrimEAA can be replicated in the Taiwanese population. Moreover, we here investigated the other three epigenetic clocks as well (Hannum et al*.*’s clock [10], Horvath’s clock [11], and Levine et al*.*’s PhenoAge [12]), and we have obtained consistent results within each generation of epigenetic clocks. Our study demonstrates that, for even CVD-free individuals, ideal CVH is important with its link to a slower aging rate in an Asian population.

## Conclusions

This is one of the first studies to comprehensively investigate the associations of CVH with four epigenetic clocks. Ideal CVH is associated with lower levels of EAA calculated according to the second-generation epigenetic clocks in an Asian population. Having an ideal CVH status can lower EAA and reduce the risk of aging-related disorders.

## Methods

### Taiwan Biobank

The TWB has recruited ~ 153,543 community-based volunteers living in Taiwan since October 2012. The written informed consent of each participant was obtained before joining the study. Participants provided blood and urine samples and then underwent physical examinations. Clinical factors of participants were examined and recorded by the TWB. For example, serum glucose was measured with a Hitachi LST008 analyzer (Hitachi High-Technologies, Tokyo, Japan) after a fast for at least 6 h. Participants’ lifestyle factors such as dietary habits, cigarette smoking, physical exercise, educational attainment, and alcohol consumption were collected through a face-to-face interview with TWB researchers.

Because responding to all items in the original TWB questionnaire was time-consuming, a simplified version of the questionnaire was developed to speed up the interview process. Diet-related questions were not included in this simplified version of the questionnaire. As a result, the diet information of 42% of the 2474 individuals was missing because 1039 participants chose the simplified questionnaire.

From 2016 to 2021, the TWB randomly selected 2474 participants for DNAm quantification analysis. The Illumina Infinium MethylationEPIC BeadChip (Illumina, Inc., San Diego, CA) was used to analyze their blood DNAm levels.

The TWB was approved by the Ethics and Governance Council of Taiwan Biobank and the Institutional Review Board on Biomedical Science Research/IRB-BM at Academia Sinica. The TWB approved our access to the data on February 18, 2020, with an application number of TWBR10810-07. Our study was also approved by the Research Ethics Committee of National Taiwan University Hospital (NTUH-REC no. 201805050RINB). Written informed consent was obtained from each individual, following the principles of the Declaration of Helsinki and the institutional requirements.

### Epigenetic age acceleration

The quality control and normalization of the DNAm data can be found in our previous works [20, 21]. Briefly speaking, the quality of all 2474 samples was satisfactory according to the quality control process of DNAm data [30]. Our intensity data were normalized with the normal-exponential out-of-band (noob) method [31] implemented by the *preprocessNoob* function in the R package minfi v1.36 [32].

To calculate epigenetic age, we uploaded the DNAm data quantified through the blood samples of the 2474 TWB participants to the online DNAm Age Calculator developed by Horvath’s laboratory (https://dnamage.genetics.ucla.edu/new). Four epigenetic clocks were applied, including Hannum et al*.*’s clock [10], Horvath’s clock [11], Levine et al*.*’s PhenoAge [12], and Lu et al*.*’s GrimAge [13]. Once the epigenetic age was calculated, four measures of EAA (HannumEAA, IEAA, PhenoEAA, and GrimEAA, respectively) were then obtained by the residuals of regressing the corresponding DNAm age on chronological age.

According to the definition of residuals, EAA is the difference between one’s DNAm age and the predicted DNAm age of individuals at the same chronological age. Positive EAA levels indicate that individuals are aging faster than the expected aging rate at the same chronological age. Contrarily, negative EAA levels imply that individuals are aging slower than the expected aging rate at the same chronological age.

### Cardiovascular health score

CVH scores were calculated according to AHA’s “Life’s simple seven” CVH metrics [1]. The seven components include smoking status, physical activity, dietary habits, BMI, total cholesterol, blood pressure, and fasting glucose (Table 3). The CVH score integrates the seven components mentioned above, representing individuals’ health in cardiovascular conditions. Two different scales were used to evaluate the idealness of each CVH component. Each component was scored as 0–1 on a 2-level scale (ideal vs. not ideal) or 0–2 on a 3-level scale (poor, intermediate, or ideal) (Table 3). Therefore, a 7-point CVH score and a 14-point CVH score were obtained by summing up all scores from the seven components.

**Table 3:** Ideal CVH criterion (the 14-point CVH score)

	Poor: 0 point^a^	Intermediate: 1 point^a^	Ideal: 2 points^a^
*Lifestyle factors*			
Smoking status	Current	Former (quit < 6 months)	Never or former^b^ (quit ≥ 6 month)
Physical activity (regular exercise)^c^	Never	Between never and regular	30 min 3 times a week (Regular exercise)
Ideal diet (Ideal diet score summed from Table S4)	0–5	6–11	12–17
*Clinical factors*			
BMI (kg/m^2^)	BMI ≥ 27	24 ≤ BMI < 27	BMI < 24
Total cholesterol (mg/dL)	TC ≥ 240	200 ≤ TC < 240	TC < 200
Blood pressure level^d^ (mmHg)	SBP ≥ 140 or DBP ≥ 90	120 ≤ SBP < 140 or 80 ≤ DBP < 90	SBP < 120 and DBP < 80
Fasting glucose (mg/dL)	Fasting glucose ≥ 126	100 ≤ Fasting glucose < 126	Fasting glucose < 100

^a^For the 7-point CVH score, each component was scored as 1 if ideal, 0 if intermediate or poor

^b^According to the TWB questionnaire, former smokers were defined as individuals who have quitted smoking for at least 6 months

^c^Regular exercise was defined as performing exercise for 30 min at least 3 times a week. The exercise included leisure-time activities such as swimming, cycling, jogging, weight training, dancing, and mountain climbing, etc.

^d^*SBP* Systolic blood pressure, *DBP* Diastolic blood pressure

A higher CVH score reflects a better overall CVH condition, associated with a lower incidence of CVD-related mortality and morbidity [33–35]. Generally, an individual’s CVH was categorized as favorable given at least 5 CVH components achieving the ideal criteria (i.e., CVH score ≥ 5 points on the 7-point scale, or CVH score ≥ 10 points on the 14-point scale); moderate given 3–4 components fulfilling the ideal criteria; unfavorable given ≤ 2 components achieving the ideal standards (i.e., CVH score ≤ 2 points on the 7-point scale, or CVH score ≤ 4 points on the 14-point scale) [36–38].

Some studies have suggested more stringent BMI cutoff levels for East Asians [39–41]. BMI > 24 has been found to be a risk factor for CVD, type 2 diabetes, and metabolic syndromes in Taiwanese populations [42, 43]. Therefore, according to the definition by the Ministry of Health and Welfare of Taiwan, BMI over 24 was defined as overweight, and BMI over 27 was regarded as obesity (Table 3).

To let “smoking status” be consistent with the TWB questionnaire, we adjusted the definition of a former smoker from quitting smoking for at least 12–6 months. Moreover, regarding “ideal diet,” because TWB did not survey the amount of various foods consumed every day, the scoring of ideal diet was modified according to 17 diet-related questions in the TWB questionnaire (Additional file 1: Table S4). Furthermore, 42% of the 2474 participants chose the simplified version of the TWB questionnaire, and therefore the diet-related information was only available for 58% of the individuals in our study. To not compromise the statistical power by only analyzing 58% of the data, we also calculated a 6-component CVH score while removing the “ideal diet” component. Therefore, we had a 6-point scale and a 12-point scale of the CVH score, following the 2-level and 3-level scoring rules, respectively.

### Exclusion criteria

Similar to the previous study linking CVH with EAA (focusing on the WHI data) [5], we excluded participants diagnosed with leukemia or CVDs and participants with extreme EAA levels. Including leukemia cases into DNAm data analysis may introduce bias [5] and reduce the precision of DNAm clock models [44]. Therefore, participants with leukemia diagnoses were excluded from our research. However, because no individuals have been diagnosed with leukemia in our study sample, we did not exclude any participants due to this criterion.

Following the World Health Organization (WHO) definition, CVDs included the diagnosis of valvular heart disease, coronary artery disease, arrhythmia, cardiomyopathy, congenital heart disease, apoplexy or any other diseases involving blood vessels or the heart. We removed 276 participants with CVDs because we aimed to test the associations of the CVH scores with EAA among Asian individuals without CVDs.

Moreover, extreme outliers were defined if EAA $> Q_{3} + 3 \times {\text{IQR}}$ or EAA $< Q_{1} - 3 \times {\text{IQR}}$, where $Q_{1}$ and $Q_{3}$ were the 25th and 75th percentiles and ${\text{IQR}} = Q_{3} - Q_{1}$ (i.e., interquartile range). Among the 2474 TWB participants, 7, 1, 2, and 5 extreme outliers were excluded from our analyses for HannumEAA, IEAA, PhenoEAA, and GrimEAA, respectively.

### Statistical analysis

To investigate the association of CVH with EAA, we regressed each of the four measures of EAA (HannumEAA [10], IEAA [11], PhenoEAA [12], and GrimEAA [13]) on the CVH score after excluding 276 participants with CVDs and participants with extreme EAA levels.

For the regression analysis, we considered the following model:

1 $${\text{EAA}} = \beta_{0} + \beta_{1} {\text{CVH}}_{{{\text{score}}}} + \beta_{2} {\text{SEX}} + \beta_{3} {\text{DRK}} + \beta_{4} {\text{EDU}} + \varepsilon ,$$

where EAA was served as the response variable, the CVH score was the primary explanatory variable, and $\varepsilon$ was the random error term. Four different calculations of the CVH scores (7-point, 14-point, 6-point, and 12-point) were included in our analyses to evaluate whether our main conclusions may be sensitive to the CVH scoring scales or the exclusion of diet information. A total of 16 regression models were therefore fitted according to four measures of EAA (IEAA, HannumEAA, PhenoEAA, and GrimEAA) and four definitions of the CVH score (6-, 7-, 12-, and 14-point scales).

Similar to the two previous studies linking CVH with EAA [5, 6], all our models were adjusted for sex (SEX), alcohol drinking status (yes vs. no), and educational attainment (an integer ranging from 1 to 7). Drinking (DRK) was defined as an individual having more than 150 mL intake of alcohol per week for at least 6 months. Educational attainment (EDU) was classified into seven categories (1: Illiterate; 2: No formal education but literate; …; 7: Master’s or higher degree), as listed in Table 1.

We considered EDU a covariate because it was associated with EAA based on various DNAm clocks [45, 46]. Moreover, EDU was also adjusted in the models of the two previous studies linking CVH with EAA [5, 6]. In our analyses, EDU was inversely associated with GrimEAA in the 6-point and 12-point CVH score models (both *p* = 0.002, Additional file 1: Table S5) and PhenoEAA in all models (*p* < 0.05, Additional file 1: Table S5).

Variance inflation factor (VIF) values were calculated (Additional file 1: Table S6) to check multicollinearity. All VIF values were controlled under 1.2 (the largest VIF value was 1.1842). No multicollinearity among the explanatory variables was detected in all our models.

Regarding the model evaluation, we performed residual analyses (Additional file 1: Figs. S1–S4) to check the assumptions of normality and constant variance. No substantial violation of these two assumptions was observed in regression models based on IEAA (Additional file 1: Fig. S1), HannumEAA (Additional file 1: Fig. S2), and PhenoEAA (Additional file 1: Fig. S3). However, the quantile–quantile plots of the models based on GrimEAA (Additional file 1: Fig. S4) showed that the residuals followed distributions with heavier tails than the normal distribution.

Deviations from normality usually do not bias the regression coefficients (Beta in Table 2) [47, 48]. To evaluate whether our statistical significance (*p*-values) may be sensitive to the violation of the normality assumption, we further performed the rank-based inverse normal transformation (rank-based INT) on GrimEAA (Additional file 1: Fig. S8). Through this transformation, no substantial violation of the assumption of normality or constant variance was observed for any model (Additional file 1: Fig. S8). Results of using the rank-based INT on the four measures of EAA as the response variable in model (1) were shown in Additional file 1: Table S7. The statistical significance was similar to our original results (Table 2).

## Supplementary Information

**Additional file 1. Table S1.** Basic characteristics of the 2474 TWB participants stratified by tertiles of PhenoEAA. **Table S2.** Basic characteristics of the 2474 TWB participants stratified by tertiles of IEAA. **Table S3.** Basic characteristics of the 2474 TWB participants stratified by tertiles of HannumEAA. **Table S4.** The 17 diet-related questions in the TWB questionnaire. **Table S5.** Regression coefficients of all the covariates included in statistical models. **Table S6.** Variance inflation factors (VIF) to check multicollinearity. **Table S7.** Regressing rank-based inverse normal transformation of the four measures of EAA on the CVH score. **Figure S1.** Residual and Normal Quantile-Quantile plots for regression models based on IEAA. **Figure S2.** Residual and Normal Quantile-Quantile plots for regression models based on HannumEAA. **Figure S3.** Residual and Normal Quantile-Quantile plots for regression models based on PhenoEAA. **Figure S4.** Residual and Normal Quantile-Quantile plots for regression models based on GrimEAA. **Figure S5.** Residual and Normal Quantile-Quantile plots for rank-based inverse normal transformation (rank-based INT) of IEAA. **Figure S6.** Residual and Normal Quantile-Quantile plots for rank-based inverse normal transformation (rank-based INT) of HannumEAA. **Figure S7.** Residual and Normal Quantile-Quantile plots for rank-based inverse normal transformation (rank-based INT) of PhenoEAA. **Figure S8.** Residual and Normal Quantile-Quantile plots for rank-based inverse normal transformation (rank-based INT) of GrimEAA.

## Data Availability

The datasets used and analyzed during the current study are available from https://www.twbiobank.org.tw/.

## Abbreviations

CVH: Cardiovascular health
AHA: American Heart Association
EAA: Epigenetic age acceleration
DNAm: DNA methylation
TWB: Taiwan Biobank
IEAA: Intrinsic EAA
EEAA: Extrinsic EAA
CVH score: Cardiovascular health score
BMI: Body mass index
CVD: Cardiovascular disease
CpG: Cytosine-phosphate-guanine
WHI: Women’s Health Initiative
CARDIA: Coronary Artery Risk Development in Young Adults
FHS: Framingham Heart Study
IRB-BM: Institutional Review Board on Biomedical Science Research
NTU: National Taiwan University
NTUH: National Taiwan University Hospital
CI: Confidence interval
SD: Standard deviation
VIF: Variance inflation factor
INT: Inverse normal transformation
WHO: World Health Organization
