# Supplementary material for: Cardiovascular health and four epigenetic clocks
Source: Clin Epigenetics. 2022 Jun 9;14:73. doi: 10.1186/s13148-022-01295-7 (PMC9185918; doi:10.1186/s13148-022-01295-7)

# Supplementary Materials

Cardiovascular health and four epigenetic clocks

Yun-Hsiang Lo ^1^, Wan-Yu Lin ^1,2,3^*

^1^ Institute of Epidemiology and Preventive Medicine, College of Public Health, National Taiwan University, Taipei, Taiwan

^2^ Master of Public Health Degree Program, College of Public Health, National Taiwan University, Taipei, Taiwan

^3^ Department of Public Health, College of Public Health, National Taiwan University, Taipei, Taiwan

Running title: Cardiovascular health and aging

* Corresponding author: Wan-Yu Lin, Ph.D.

Wan-Yu Lin, Ph.D. (<http://orcid.org/0000-0002-3385-4702>)

Room 501, No. 17, Xu-Zhou Road, Taipei 100, Taiwan

Phone/Fax: +886-2-33668106; E-mail: [linwy@ntu.edu.tw](mailto:linwy@ntu.edu.tw)

Supplemental Tables

Table S1: Basic characteristics of the 2,474 TWB participants stratified by tertiles of PhenoEAA

Table S2: Basic characteristics of the 2,474 TWB participants stratified by tertiles of IEAA

Table S3: Basic characteristics of the 2,474 TWB participants stratified by tertiles of HannumEAA

Table S4: The 17 diet-related questions in the TWB questionnaire

Table S5: Regression coefficients of all the covariates included in statistical models

Table S6: Variance inflation factors (VIF) to check multicollinearity

Table S7: Regressing rank-based inverse normal transformation of the four measures of EAA on the CVH score

Supplemental Figures

Figure S1: Residual and Normal Quantile-Quantile plots for regression models based on IEAA

Figure S2: Residual and Normal Quantile-Quantile plots for regression models based on HannumEAA

Figure S3: Residual and Normal Quantile-Quantile plots for regression models based on PhenoEAA

Figure S4: Residual and Normal Quantile-Quantile plots for regression models based on GrimEAA

Figure S5: Residual and Normal Quantile-Quantile plots for rank-based inverse normal transformation (rank-based INT) of IEAA

Figure S6: Residual and Normal Quantile-Quantile plots for rank-based inverse normal transformation (rank-based INT) of HannumEAA

Figure S7: Residual and Normal Quantile-Quantile plots for rank-based inverse normal transformation (rank-based INT) of PhenoEAA

Figure S8: Residual and Normal Quantile-Quantile plots for rank-based inverse normal transformation (rank-based INT) of GrimEAA

| **Table S1. Basic characteristics of the 2,474 TWB participants stratified by tertiles of PhenoEAA** | | | | | | | | |
| --- | --- | --- | --- | --- | --- | --- | --- | --- |
|  |  | Overall |  | PhenoEAA T1  (< -2.16 years.)**^Ref^** |  | PhenoEAA T2  (-2.16~1.89 years.) |  | PhenoEAA T3  (> 1.89 years.) |
| *N* (male %) |  | 2474 (50.24%) |  | 825 (49.70%) |  | 824 (49.27%) |  | 825 (51.76%) |
| *Chronological age (standard deviation, s.d.)* |  | 49.76 (11.08) |  | 50.01 (11.72) |  | 49.41 (10.68) |  | 49.85 (10.81) |
| *Education (%)* |  |  |  |  |  |  |  |  |
| Illiterate |  | 5 (0.20%) |  | 1 (0.12%) |  | 1 (0.12%) |  | 3 (0.36%) |
| No formal education but literate |  | 2 (0.08%) |  | 0 (0.00%) |  | 2 (0.24%) |  | 0 (0.00%) |
| Primary school graduate |  | 95 (3.84%) |  | 32 (3.88%) |  | 27 (3.28%) |  | 36 (4.36%) |
| Junior high school graduate |  | 137 (5.54%) |  | 36 (4.36%) |  | 48 (5.83%) |  | 53 (6.42%) |
| Senior high school graduate |  | 718 (29.02%) |  | 230 (27.88%) |  | 244 (29.61%) |  | 244 (29.58%) |
| College graduate |  | 1254 (50.69%) |  | 430 (52.12%) |  | 410 (49.76%) |  | 414 (50.18%) |
| Master’s or higher degree |  | 261 (10.55%) |  | 96 (11.64%) |  | 90 (10.92%) |  | 75 (9.09%) |
| *7 components of the CVH score (%)* |  |  |  |  |  |  |  |  |
| Smoking status – never |  | 1614 (65.24%) |  | 570 (69.09%) |  | 527 (63.88%) * |  | 517 (62.67%) ** |
| Smoking status – former^1^ |  | 312 (12.61%) |  | 92 (11.15%) |  | 96 (11.64%) |  | 124 (15.03%) * |
| Smoking status – current |  | 283 (11.44%) |  | 69 (8.36%) |  | 109 (13.21%) ** |  | 105 (12.73%) ** |
| Ideal BMI^2^ |  | 1240 (50.12%) |  | 472 (57.21%) |  | 395 (47.88%) *** |  | 373 (45.21%) *** |
| Ideal physical activity^3^ |  | 1092 (44.14%) |  | 409 (49.58%) |  | 349 (42.30%) ** |  | 334 (40.48%) *** |
| Ideal cholesterol level^4^ |  | 1444 (58.37%) |  | 453 (54.91%) |  | 488 (59.15%) |  | 503 (60.97%) * |
| Ideal fasting glucose level^5^ |  | 1951 (78.86%) |  | 671 (81.33%) |  | 642 (77.82%) |  | 638 (77.33%) |
| Ideal blood pressure^6^ |  | 1315 (53.15%) |  | 461 (55.88%) |  | 450 (54.55%) |  | 404 (48.97%) ** |
| Ideal diet^7^ |  | 456 (31.78% out of 1435) |  | 167 (34.08% out of 490) |  | 146 (30.10% out of 485) |  | 143 (31.09% out of 460) |
| *6-point CVH score (%)* |  |  |  |  |  |  |  |  |
| *N* |  | 2471 |  | 824 |  | 823 |  | 824 |
| 0-1 |  | 127 (5.13%) |  | 23 (2.79%) |  | 52 (6.30%) *** |  | 52 (6.30%) *** |
| 2 |  | 329 (13.30%) |  | 98 (11.88%) |  | 110 (13.33%) |  | 121 (14.67%) |
| 3 |  | 554 (22.39%) |  | 158 (19.15%) |  | 180 (21.82%) |  | 216 (26.18%) *** |
| 4 |  | 716 (28.94%) |  | 268 (32.48%) |  | 246 (29.82%) |  | 202 (24.48%) *** |
| 5 |  | 586 (23.69%) |  | 219 (26.55%) |  | 181 (21.94%) * |  | 186 (22.55%) |
| 6 |  | 159 (6.43%) |  | 58 (7.04%) |  | 54 (6.56%) |  | 47 (5.70%) |

^Ref^ T1 (tertile 1, the reference group); T2 (or T3) compared with T1 based on the two-sample proportion test; **p* < 0.05; ** *p*< 0.01; *** *p* < 0.001.

^1^A former smoker was defined as an individual who has quitted smoking for at least 6 months.

^2^Ideal BMI: body mass index less than 24 kg/m^2^, according to the criterion proposed by the Ministry of Health and Welfare, Taiwan.

^3^Ideal physical activity was defined as performing 30 minutes of exercise (including leisure-time activities such as swimming, cycling, jogging, weight training, dancing, mountain climbing, etc.) at least 3 times a week.

^4^Ideal cholesterol level was defined as total cholesterol level less than 200 mg/dL.

^5^Ideal fasting glucose level was defined as fasting glucose level less than 100 mg/dL.

^6^Ideal blood pressure was defined as systolic blood pressure less than 120 mmHg and diastolic blood pressure below 80 mmHg.

^7^Ideal diet was assessed according to the consumption of food categories, sodium and fat intake.

| **Table S2. Basic characteristics of the 2,474 TWB participants stratified by tertiles of IEAA** | | | | | | | | |
| --- | --- | --- | --- | --- | --- | --- | --- | --- |
|  |  | Overall |  | IEAA T1  (< -1.60 years.)**^Ref^** |  | IEAA T2  (-1.60~1.48 years.) |  | IEAA T3  (> 1.48 years.) |
| *N* (male %) |  | 2474 (50.24%) |  | 825 (40.24%) |  | 824 (49.88%) *** |  | 825 (60.61%) *** |
| *Chronological age (standard deviation, s.d.)* |  | 49.76 (11.08) |  | 50.14 (11.79) |  | 49.34 (11.14) |  | 49.79 (10.25) |
| *Education (%)* |  |  |  |  |  |  |  |  |
| Illiterate |  | 5 (0.20%) |  | 1 (0.12%) |  | 3 (0.36%) |  | 1 (0.12%) |
| No formal education but literate |  | 2 (0.08%) |  | 2 (0.24%) |  | 0 (0.00%) |  | 0 (0.00%) |
| Primary school graduate |  | 95 (3.84%) |  | 40 (4.85%) |  | 33 (4.00%) |  | 22 (2.67%) * |
| Junior high school graduate |  | 137 (5.54%) |  | 48 (5.82%) |  | 39 (4.73%) |  | 50 (6.06%) |
| Senior high school graduate |  | 718 (29.02%) |  | 239 (28.97%) |  | 238 (28.88%) |  | 241 (29.21%) |
| College graduate |  | 1254 (50.69%) |  | 410 (49.70%) |  | 431 (52.31%) |  | 413 (50.06%) |
| Master’s or higher degree |  | 261 (10.55%) |  | 85 (10.30%) |  | 80 (9.71%) |  | 96 (11.64%) |
| *7 components of the CVH score (%)* |  |  |  |  |  |  |  |  |
| Smoking status – never |  | 1614 (65.24%) |  | 596 (72.24%) |  | 534 (64.73%) ** |  | 484 (58.67%) *** |
| Smoking status – former^1^ |  | 312 (12.61%) |  | 83 (10.06%) |  | 104 (12.61%) |  | 125 (15.15%) ** |
| Smoking status – current |  | 283 (11.44%) |  | 76 (9.21%) |  | 94 (11.39%) |  | 113 (13.70%) ** |
| Ideal BMI^2^ |  | 1240 (50.12%) |  | 437 (52.97%) |  | 428 (51.88%) |  | 375 (45.45%) ** |
| Ideal physical activity^3^ |  | 1092 (44.14%) |  | 376 (45.58%) |  | 354 (42.91%) |  | 362 (43.88%) |
| Ideal cholesterol level^4^ |  | 1444 (58.37%) |  | 455 (55.15%) |  | 494 (59.88%) |  | 495 (60.00%) |
| Ideal fasting glucose level^5^ |  | 1951 (78.86%) |  | 656 (79.52%) |  | 657 (79.64%) |  | 638 (77.33%) |
| Ideal blood pressure^6^ |  | 1315 (53.15%) |  | 469 (56.85%) |  | 450 (54.55%) |  | 396 (48.00%) *** |
| Ideal diet^7^ |  | 456 (31.78% out of 1435) |  | 155 (32.36% out of 479) |  | 155 (31.00% out of 500) |  | 146 (32.02% out of 456) |
| *6-point CVH score (%****^e^*** |  |  |  |  |  |  |  |  |
| *N* |  | 2471 |  | 824 |  | 823 |  | 824 |
| 0-1 |  | 127 (5.13%) |  | 32 (3.88%) |  | 41 (4.97%) |  | 54 (6.55%) * |
| 2 |  | 329 (13.30%) |  | 98 (11.88%) |  | 104 (12.61%) |  | 127 (15.39%) * |
| 3 |  | 554 (22.39%) |  | 173 (20.97%) |  | 190 (23.03%) |  | 191 (23.15%) |
| 4 |  | 716 (28.94%) |  | 262 (31.76%) |  | 228 (27.64%) |  | 226 (27.39%) |
| 5 |  | 586 (23.69%) |  | 216 (26.18%) |  | 192 (23.27%) |  | 178 (21.58%) * |
| 6 |  | 159 (6.43%) |  | 43 (5.22%) |  | 68 (8.26%) * |  | 48 (5.83%) |

^Ref^ T1 (tertile 1, the reference group); T2 (or T3) compared with T1 based on the two-sample proportion test; **p* < 0.05; ** *p*< 0.01; *** *p* < 0.001.

^1^A former smoker was defined as an individual who has quitted smoking for at least 6 months.

^2^Ideal BMI: body mass index less than 24 kg/m^2^, according to the criterion proposed by the Ministry of Health and Welfare, Taiwan.

^3^Ideal physical activity was defined as performing 30 minutes of exercise (including leisure-time activities such as swimming, cycling, jogging, weight training, dancing, mountain climbing, etc.) at least 3 times a week.

^4^Ideal cholesterol level was defined as total cholesterol level less than 200 mg/dL.

^5^Ideal fasting glucose level was defined as fasting glucose level less than 100 mg/dL.

^6^Ideal blood pressure was defined as systolic blood pressure less than 120 mmHg and diastolic blood pressure below 80 mmHg.

^7^Ideal diet was assessed according to the consumption of food categories, sodium and fat intake.

| **Table S3. Basic characteristics of the 2,474 TWB participants stratified by tertiles of HannumEAA** | | | | | | | | |
| --- | --- | --- | --- | --- | --- | --- | --- | --- |
|  |  | Overall |  | HannumEAA T1  (< -1.58 years.)**^Ref^** |  | HannumEAA T2  (-1.58~1.37 years.) |  | HannumEAA T3  (> 1.37 years.) |
| *N* (male %) |  | 2474 (50.24%) |  | 825 (38.79%) |  | 824 (50.61%) *** |  | 825 (61.33%) *** |
| *Chronological age (standard deviation, s.d.)* |  | 49.76 (11.08) |  | 50.08 (11.53) |  | 49.21 (10.86) |  | 49.98 (10.82) |
| *Education (%)* |  |  |  |  |  |  |  |  |
| Illiterate |  | 5 (0.20%) |  | 3 (0.36%) |  | 1 (0.12%) |  | 1 (0.12%) |
| No formal education but literate |  | 2 (0.08%) |  | 0 (0.00%) |  | 0 (0.00%) |  | 2 (0.24%) |
| Primary school graduate |  | 95 (3.84%) |  | 29 (3.52%) |  | 38 (4.61%) |  | 28 (3.39%) |
| Junior high school graduate |  | 137 (5.54%) |  | 45 (5.45%) |  | 51 (6.19%) |  | 41 (4.97%) |
| Senior high school graduate |  | 718 (29.02%) |  | 244 (29.58%) |  | 233 (28.28%) |  | 241 (29.21%) |
| College graduate |  | 1254 (50.69%) |  | 418 (50.67%) |  | 411 (49.88%) |  | 425 (51.52%) |
| Master’s or higher degree |  | 261 (10.55%) |  | 86 (10.42%) |  | 90 (10.92%) |  | 85 (10.30%) |
| *7 components of the CVH score (%)* |  |  |  |  |  |  |  |  |
| Smoking status – never |  | 1614 (65.24%) |  | 593 (71.88%) |  | 546 (66.18%) * |  | 475 (57.58%) *** |
| Smoking status – former^1^ |  | 312 (12.61%) |  | 83 (10.06%) |  | 93 (11.27%) |  | 136 (16.48%) *** |
| Smoking status – current |  | 283 (11.44%) |  | 70 (8.48%) |  | 97 (11.76%) * |  | 116 (14.06%) *** |
| Ideal BMI^2^ |  | 1240 (50.12%) |  | 457 (55.39%) |  | 402 (48.73%) ** |  | 381 (46.18%) *** |
| Ideal physical activity^3^ |  | 1092 (44.14%) |  | 373 (45.21%) |  | 350 (42.42%) |  | 369 (44.73%) |
| Ideal cholesterol level^4^ |  | 1444 (58.37%) |  | 452 (54.79%) |  | 494 (59.88%) * |  | 498 (60.36%) * |
| Ideal fasting glucose level^5^ |  | 1951 (78.86%) |  | 677 (82.06%) |  | 649 (78.67%) |  | 625 (75.76%) ** |
| Ideal blood pressure^6^ |  | 1315 (53.15%) |  | 479 (58.06%) |  | 446 (54.06%) |  | 390 (47.27%) *** |
| Ideal diet^7^ |  | 456 (31.78% out of 1435) |  | 178 (35.53% out of 501) |  | 142 (29.71% out of 478) |  | 136 (29.82% out of 456) |
| *6-point CVH score (%)* |  |  |  |  |  |  |  |  |
| *N* |  | 2471 |  | 824 |  | 823 |  | 824 |
| 0-1 |  | 127 (5.13%) |  | 34 (4.12%) |  | 44 (5.33%) |  | 49 (5.94%) |
| 2 |  | 329 (13.30%) |  | 93 (11.27%) |  | 112 (13.58%) |  | 124 (15.03%) * |
| 3 |  | 554 (22.39%) |  | 165 (20.00%) |  | 179 (21.7%) |  | 210 (25.45%) ** |
| 4 |  | 716 (28.94%) |  | 251 (30.42%) |  | 240 (29.09%) |  | 225 (27.27%) |
| 5 |  | 586 (23.69%) |  | 227 (27.52%) |  | 199 (24.12%) |  | 160 (19.39%) *** |
| 6 |  | 159 (6.43%) |  | 54 (6.55%) |  | 49 (5.95%) |  | 56 (6.80%) |

^Ref^ T1 (tertile 1, the reference group); T2 (or T3) compared with T1 based on the two-sample proportion test; **p* < 0.05; ** *p*< 0.01; *** *p* < 0.001.

^1^A former smoker was defined as an individual who has quitted smoking for at least 6 months.

^2^Ideal BMI: body mass index less than 24 kg/m^2^, according to the criterion proposed by the Ministry of Health and Welfare, Taiwan.

^3^Ideal physical activity was defined as performing 30 minutes of exercise (including leisure-time activities such as swimming, cycling, jogging, weight training, dancing, mountain climbing, etc.) at least 3 times a week.

^4^Ideal cholesterol level was defined as total cholesterol level less than 200 mg/dL.

^5^Ideal fasting glucose level was defined as fasting glucose level less than 100 mg/dL.

^6^Ideal blood pressure was defined as systolic blood pressure less than 120 mmHg and diastolic blood pressure below 80 mmHg.

^7^Ideal diet was assessed according to the consumption of food categories, sodium and fat intake.

| **Table S4. The 17 diet-related questions in the TWB questionnaire** | | | | | |
| --- | --- | --- | --- | --- | --- |
| Dietary habits/ Food Category | Always | Most of the time | Half of the time | Seldom | Never |
| 1. When you eat meat (such as pork, beef, mutton, chicken, duck, goose, etc.), do you eat it with fat, suet, or skin? | 1 | 2 | 3 | 4 | 5 |
| 1. When you eat fish or meat, do you prefer cooking it with oil (such as frying, deep-frying, frying and then braised, steamed fish topped with oil, etc.)? | 1 | 2 | 3 | 4 | 5 |
| 1. When you eat vegetables, do you prefer cooking them in stir-fry way? | 1 | 2 | 3 | 4 | 5 |
| 1. When you eat rice or noodles (staple food), do you eat them with marinade, gravy, or lard? | 1 | 2 | 3 | 4 | 5 |
| 1. When you eat soy foods, do you prefer cooking them in deep-fry way (such as fried tofu, stinky tofu, fried tofu skin, etc.)? | 1 | 2 | 3 | 4 | 5 |
| 1. When you eat bread, do you spread butter, plant-based butter (margarine), or mayonnaise? | 1 | 2 | 3 | 4 | 5 |
| 1. When you have a meal, do you add additional salt, soy sauce, chili sauce, or any other seasoning? | 1 | 2 | 3 | 4 | 5 |
| 1. Are you used to having pickles, fermented tofu, fermented soy beans as side dishes in a meal? | 1 | 2 | 3 | 4 | 5 |
| 1. When you have snacks, do you choose to eat fruits or vegetables instead of high-fat snacks (such as chips, cakes, doughnuts, etc.)? | 1 | 2 | 3 | 4 | 5 |
| 1. When you prepare meat (such as pork, beef, mutton, chicken, duck, goose, etc.) for a meal, do you cook it in roasted or braised way instead of deep-frying? | 1 | 2 | 3 | 4 | 5 |
| 1. If a food product has a low-fat option (such as low-fat ice cream, low-fat milk, skim milk, low-fat salad sauce, etc.), would you choose it instead of regular product? | 1 | 2 | 3 | 4 | 5 |
| 1. Do you eat food with low-sodium ingredients (such as low-sodium salt, lower-sodium soy sauce, etc.)? | 1 | 2 | 3 | 4 | 5 |
| 1. Would you like to eat lower-fat meat (such as fish or chicken) instead of higher-fat meat (such as beef or pork)? | 1 | 2 | 3 | 4 | 5 |
| 1. Would you choose to eat lean meat instead of fatty meat? | 1 | 2 | 3 | 4 | 5 |
| 1. Would you choose to perform vegetarian and light diet in certain meals of the day, to reduce the intake of higher-fat food such as meat or fat. | 1 | 2 | 3 | 4 | 5 |
| 1. Do you eat at least 2 kinds of vegetables a day? | 1 | 2 | 3 | 4 | 5 |
| 1. When you have meat, do you intentionally eat less? | 1 | 2 | 3 | 4 | 5 |

Scoring for dietary habits:

For questions 1-8, 1 point for answering = 4 or 5; for questions 9-17, 1 point for answering = 1 or 2.

Ideal diet score was calculated by summing the scores of these 17 questions. Therefore, the ideal diet score ranged from 0 to 17. We further categorized dietary habits as poor (0-5), intermediate (6-11), and ideal (12-17), as listed in Table 3.

| **Table S5. Regression coefficients of all the covariates included in statistical models (****p* < 0.05; ** *p*< 0.01; *** *p* < 0.001**)** | | | | | | | | | | | | | | | |
| --- | --- | --- | --- | --- | --- | --- | --- | --- | --- | --- | --- | --- | --- | --- | --- |
|  | **1^st^ Generation of Epigenetic Clocks** | | | | | | |  | **2^nd^ Generation of Epigenetic Clocks** | | | | | | |
|  | **IEAA** | | |  | **HannumEAA** | | |  | **PhenoEAA** | | |  | **GrimEAA** | | |
| **Covariates** | **beta** | **95% C.I.** | ***p*** |  | **beta** | **95% C.I.** | ***p*** |  | **beta** | **95% C.I.** | ***p*** |  | **beta** | **95% C.I.** | ***P*** |
| ***Intercept*** | 1.711 | [-0.0689,  3.4902] | 0.060 |  | 1.801 | [-0.0049,  3.6076] | 0.051 |  | 2.283 | [-0.0895,  4.6554] | 0.059 |  | 5.529 | [4.0342,  7.0247] | 6.9E-13 *** |
| ***CVH score***  ***(2-level, 7 point)*** | -0.101 | [-0.2475,  0.0458] | 0.177 |  | -0.122 | [-0.2709,  0.0269] | 0.108 |  | -0.350 | [-0.5459,  -0.1550] | 4.5E-4 *** |  | -0.499 | [-0.6222,  -0.3758] | 4.2E-15 *** |
| ***SEX***  **(female vs. male)** | -1.001 | [-1.4263,  -0.5763] | 4.2E-6 *** |  | -1.161 | [-1.5925,  -0.7293] | 1.5E-7 *** |  | 0.116 | [-0.4507,  0.6825] | 0.688 |  | -2.387 | [-2.7445,  -2.0302] | 5.9E-37 *** |
| ***Drinking status***  **(Yes vs. no)** | 0.299 | [-0.5156,  1.1141] | 0.471 |  | 0.106 | [-0.7208,  0.9327] | 0.802 |  | 0.453 | [-0.6328,  1.5398] | 0.413 |  | 0.647 | [-0.0405,  1.335] | 0.065 |
| ***Educational***  ***attainment*** | -0.035 | [-0.2489,  0.179] | 0.749 |  | 0.036 | [-0.1812,  0.2534] | 0.745 |  | -0.297 | [-0.5819,  -0.0114] | 0.042 * |  | -0.120 | [-0.3000,  0.0595] | 0.190 |
|  | **1^st^ Generation of Epigenetic Clocks** | | | | | | |  | **2^nd^ Generation of Epigenetic Clocks** | | | | | | |
|  | **IEAA** | | |  | **HannumEAA** | | |  | **PhenoEAA** | | |  | **GrimEAA** | | |
| **Covariates** | **beta** | **95% C.I.** | ***P*** |  | **beta** | **95% C.I.** | ***p*** |  | **beta** | **95% C.I.** | ***P*** |  | **beta** | **95% C.I.** | ***P*** |
| ***Intercept*** | 2.078 | [0.2063,  3.9502] | 0.030 * |  | 2.136 | [0.236,  4.0364] | 0.028 * |  | 3.475 | [0.9843,  5.9666] | 6.3E-3 ** |  | 7.116 | [5.5564,  8.6761] | 1.2E-18 *** |
| ***CVH score***  ***(3-level, 14 point)*** | -0.074 | [-0.1670,  0.0194] | 0.120 |  | -0.083 | [-0.1773,  0.0119] | 0.087 |  | -0.268 | [-0.3919,  -0.1439] | 2.4E-5 *** |  | -0.364 | [-0.4419,  -0.2865] | 1.5E-19 *** |
| ***SEX***  **(female vs. male)** | -0.991 | [-1.4113,  -0.5703] | 4.2E-6 *** |  | -1.167 | [-1.5944,  -0.7401] | 9.8E-8 *** |  | 0.138 | [-0.422,  0.6972] | 0.630 |  | -2.376 | [-2.7261,  -2.0251] | 7.0E-38 *** |
| ***Drinking status***  **(Yes vs. no)** | 0.283 | [-0.5313,  1.0983] | 0.495 |  | 0.099 | [-0.728,  0.9259] | 0.814 |  | 0.408 | [-0.6763,  1.4923] | 0.461 |  | 0.595 | [-0.0868,  1.2775] | 0.087 |
| ***Educational***  ***attainment*** | -0.033 | [-0.2465,  0.1797] | 0.758 |  | 0.033 | [-0.1833,  0.2497] | 0.764 |  | -0.295 | [-0.5786,  -0.0115] | 0.041 * |  | -0.123 | [-0.3004,  0.0547] | 0.175 |
|  | **1^st^ Generation of Epigenetic Clocks** | | | | | | |  | **2^nd^ Generation of Epigenetic Clocks** | | | | | | |
|  | **IEAA** | | |  | **HannumEAA** | | |  | **PhenoEAA** | | |  | **GrimEAA** | | |
| **Covariates** | **beta** | **95% C.I.** | ***P*** |  | **beta** | **95% C.I.** | ***p*** |  | **beta** | **95% C.I.** | ***P*** |  | **beta** | **95% C.I.** | ***p*** |
| ***Intercept*** | 2.046 | [0.643,  3.4485] | 4.3E-3 ** |  | 2.351 | [0.9732,  3.7281] | 8.3E-4 ** |  | 2.110 | [0.234,  3.9869] | 0.028 * |  | 5.686 | [4.5078,  6.8638] | 7.3E-21*** |
| ***CVH score***  ***(2-level, 6 point)*** | -0.086 | [-0.2093,  0.0366] | 0.169 |  | -0.088 | [-0.2092,  0.0324] | 0.151 |  | -0.388 | [-0.5528,  -0.2238] | 3.9E-6 *** |  | -0.526 | [-0.6289,  -0.4222] | 6.1E-23*** |
| ***SEX***  **(female vs. male)** | -1.331 | [-1.6536,  -1.0078] | 1.0E-15 *** |  | -1.273 | [-1.5906,  -0.956] | 5.5E-15 *** |  | 0.187 | [-0.2454,  0.6185] | 0.397 |  | -2.397 | [-2.6684,  -2.1261] | 3.3E-63*** |
| ***Drinking status***  **(Yes vs. no)** | 0.423 | [-0.1795,  1.0254] | 0.169 |  | 0.289 | [-0.3029,  0.88] | 0.339 |  | 0.543 | [-0.2626,  1.3491] | 0.186 |  | 1.009 | [0.5006,  1.5171] | 1.0E-4*** |
| ***Educational***  ***attainment*** | -0.042 | [-0.2118,  0.1277] | 0.627 |  | -0.082 | [-0.2486,  0.085] | 0.336 |  | -0.278 | [-0.5047,  -0.0506] | 0.017 * |  | -0.231 | [-0.3733,  -0.0883] | 0.002 ** |
|  | **1^st^ Generation of Epigenetic Clocks** | | | | | | |  | **2^nd^ Generation of Epigenetic Clocks** | | | | | | |
|  | **IEAA** | | |  | **HannumEAA** | | |  | **PhenoEAA** | | |  | **GrimEAA** | | |
| **Covariates** | **beta** | **95% C.I.** | ***P*** |  | **beta** | **95% C.I.** | ***P*** |  | **beta** | **95% C.I.** | ***P*** |  | **beta** | **95% C.I.** | ***P*** |
| ***Intercept*** | 2.290 | [0.8294,  3.7515] | 2.1E-3 ** |  | 2.570 | [1.1357,  4.0049] | 4.5E-4 ** |  | 3.038 | [1.0861,  4.9896] | 2.3E-3 ** |  | 6.950 | [5.7324,  8.1677] | 2.6E-28 *** |
| ***CVH score***  ***(3-level, 12 point)*** | -0.068 | [-0.1440,  0.0085] | 0.082 |  | -0.065 | [-0.1396,  0.0102] | 0.091 |  | -0.278 | [-0.3798,  -0.1761] | 9.6E-8 *** |  | -0.377 | [-0.4407,  -0.3136] | 2.1E-30 *** |
| ***SEX***  **(female vs. male)** | -1.325 | [-1.645,  -1.0046] | 8.1E-16 *** |  | -1.272 | [-1.5862,  -0.9569] | 3.6E-15 *** |  | 0.188 | [-0.2398,  0.6156] | 0.389 |  | -2.394 | [-2.6612,  -2.1276] | 6.1E-65 *** |
| ***Drinking status***  **(Yes vs. no)** | 0.410 | [-0.192,  1.0128] | 0.182 |  | 0.280 | [-0.3118,  0.8712] | 0.354 |  | 0.509 | [-0.2958,  1.3136] | 0.215 |  | 0.959 | [0.4550,  1.4639] | 2.0E-4 *** |
| ***Educational***  ***attainment*** | -0.039 | [-0.2082,  0.131] | 0.655 |  | -0.080 | [-0.2463,  0.0872] | 0.350 |  | -0.269 | [-0.496,  -0.0428] | 0.020 * |  | -0.220 | [-0.3609,  -0.0782] | 0.002 ** |

| **Table S6. Variance inflation factors (VIF) to check multicollinearity.** VIF scores of all the explanatory variables were controlled under 1.2, indicating no multicollinearity in all models. Sex, drinking status, and educational attainment have been adjusted in all models. | | | | |
| --- | --- | --- | --- | --- |
|  | **Explanatory variables** | | | |
| **Regression Model** | **CVH score** | **SEX** | **Drinking status** | **Educational attainment** |
| **Regressing IEAA on CVH score (7-point)** | 1.1374 | 1.1842 | 1.0520 | 1.0573 |
| **Regressing IEAA on CVH score (14-point)** | 1.1105 | 1.1604 | 1.0529 | 1.0494 |
| **Regressing IEAA on CVH score (6-point)** | 1.1051 | 1.1515 | 1.0536 | 1.0524 |
| **Regressing IEAA on CVH score (12-point)** | 1.0865 | 1.1328 | 1.0540 | 1.0514 |
|  | **Explanatory variables** | | | |
| **Regression Model** | **CVH score** | **SEX** | **Drinking status** | **Educational attainment** |
| **Regressing HannumEAA on CVH score (7-point)** | 1.1374 | 1.1836 | 1.0520 | 1.0567 |
| **Regressing HannumEAA on CVH score (14-point)** | 1.1107 | 1.1599 | 1.0528 | 1.0490 |
| **Regressing HannumEAA on CVH score (6-point)** | 1.1053 | 1.1509 | 1.0536 | 1.0518 |
| **Regressing HannumEAA on CVH score (12-point)** | 1.0868 | 1.1325 | 1.0540 | 1.0508 |
|  | **Explanatory variables** | | | |
| **Regression Model** | **CVH score** | **SEX** | **Drinking status** | **Educational attainment** |
| **Regressing PhenoEAA on CVH score (7-point)** | 1.1374 | 1.1842 | 1.0520 | 1.0573 |
| **Regressing PhenoEAA on CVH score (14-point)** | 1.1105 | 1.1604 | 1.0529 | 1.0494 |
| **Regressing PhenoEAA on CVH score (6-point)** | 1.1051 | 1.1515 | 1.0536 | 1.0524 |
| **Regressing PhenoEAA on CVH score (12-point)** | 1.0865 | 1.1328 | 1.0540 | 1.0514 |
|  | **Explanatory variables** | | | |
| **Regression Model** | **CVH score** | **SEX** | **Drinking status** | **Educational attainment** |
| **Regressing GrimEAA on CVH score (7-point)** | 1.1377 | 1.1841 | 1.0509 | 1.0577 |
| **Regressing GrimEAA on CVH score (14-point)** | 1.1106 | 1.1602 | 1.0517 | 1.0498 |
| **Regressing GrimEAA on CVH score (6-point)** | 1.1047 | 1.1512 | 1.0520 | 1.0528 |
| **Regressing GrimEAA on CVH score (12-point)** | 1.0863 | 1.1324 | 1.0526 | 1.0516 |

| Table S7. Regressing rank-based inverse normal transformation of the four measures of EAA on the CVH score | | | | | | | | | | | | | | | | | | | | | | | |
| --- | --- | --- | --- | --- | --- | --- | --- | --- | --- | --- | --- | --- | --- | --- | --- | --- | --- | --- | --- | --- | --- | --- | --- |
|  | 1^st^ Generation of Epigenetic Clocks | | | | | | | | | | |  | 2^nd^ Generation of Epigenetic Clocks | | | | | | | | | | |
|  | IEAA^1^ | | | | |  | HannumEAA^1^ | | | | |  | PhenoEAA^1^ | | | | |  | GrimEAA^1^ | | | | |
|  | beta |  | 95% C.I. |  | *p* |  | beta |  | 95% C.I. |  | *p* |  | beta |  | 95% C.I. |  | *p* |  | beta |  | 95% C.I. |  | *p* |
| Seven-component CVH scores^2^ | | | | | | | | | | | | | | | | | | | | | | | |
| CVH score  (2-level, 7-point) | -0.028 |  | [-0.0675, 0.0118] |  | 0.168 |  | -0.038 |  | [-0.0784, 0.003] |  | 0.069 |  | -0.075 |  | [-0.1160, -0.0345] |  | 3.1E-4 |  | -0.138 |  | [-0.1742, -0.1018] |  | 1.4E-13 |
| CVH score  (3-level, 14-point) | -0.022 |  | [-0.0473, 0.0024] |  | 0.077 |  | -0.026 |  | [-0.0519, -0.0009] |  | 0.043 |  | -0.057 |  | [-0.0825, -0.0314] |  | 1.3E-5 |  | -0.100 |  | [-0.1228, -0.0776] |  | 8.9E-18 |
| Six-component CVH scores^3^ | | | | | | | | | | | | | | | | | | | | | | | |
| CVH score  (2-level, 6-point) | -0.025 |  | [-0.0587, 0.0081] |  | 0.137 |  | -0.025 |  | [-0.0583, 0.0082] |  | 0.139 |  | -0.082 |  | [-0.1162, -0.0478] |  | 2.8E-6 |  | -0.143 |  | [-0.1736, -0.1131] |  | 3.5E-20 |
| CVH score  (3-level, 12-point) | -0.019 |  | [-0.0399, 0.0015] |  | 0.069 |  | -0.019 |  | [-0.0392, 0.002] |  | 0.077 |  | -0.058 |  | [-0.0794, -0.0371] |  | 7.6E-8 |  | -0.102 |  | [-0.1206, -0.0834] |  | 3.1E-26 |

^1^IEAA (i.e., intrinsic EAA), HannumEAA, PhenoEAA, and GrimEAA were calculated according to the four epigenetic clocks: Horvath’s clock [11], Hannum et al’s clock [10], Levine et al’s PhenoAge [12], and Lu et al’s GrimAge [13], respectively.

^2^ The seven-component CVH score was calculated according to the definition of CVH from the American Heart Association (AHA).

^3^ Because 42% of the 2,474 TWB participants were surveyed by the simplified questionnaire without diet information, the six-component CVH score was calculated without the “ideal diet score”. Other components followed the same definition of the CVH score from the AHA.

**Figure S1. Residual and Normal Quantile-Quantile plots for regression models based on IEAA.** (A-D) Residual plots for regressing IEAA on: (A) 6-point CVH score; (B) 7-point CVH score; (C) 12-point CVH score; (D) 14-point CVH score. (E-H) The Normal Q-Q plots for models regressing IEAA on: (E) 6-point CVH score; (F) 7-point CVH score; (G) 12-point CVH score; (H) 14-point CVH score. Sex, drinking status, and educational attainment have been adjusted in all models. No substantial violation of the assumption of normality or constant variance was observed for any model.

**
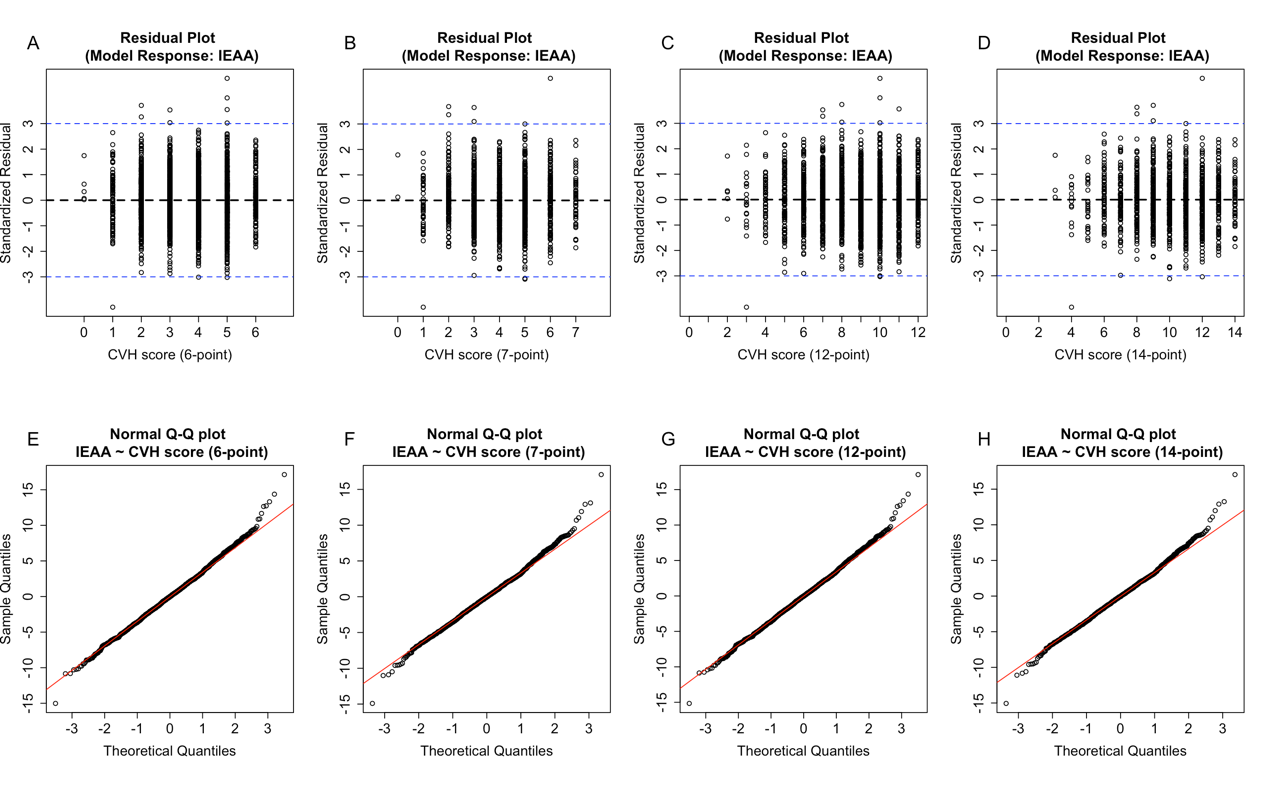
**

**Figure S2. Residual and Normal Quantile-Quantile plots for regression models based on HannumEAA.** (A-D) Residual plots for regressing HannumEAA on: (A) 6-point CVH score; (B) 7-point CVH score; (C) 12-point CVH score; (D) 14-point CVH score. (E-H) The Normal Q-Q plots for models regressing HannumEAA on: (E) 6-point CVH score; (F) 7-point CVH score; (G) 12-point CVH score; (H) 14-point CVH score. Sex, drinking status, and educational attainment have been adjusted in all models. No substantial violation of the assumption of normality or constant variance was observed for any model.

**
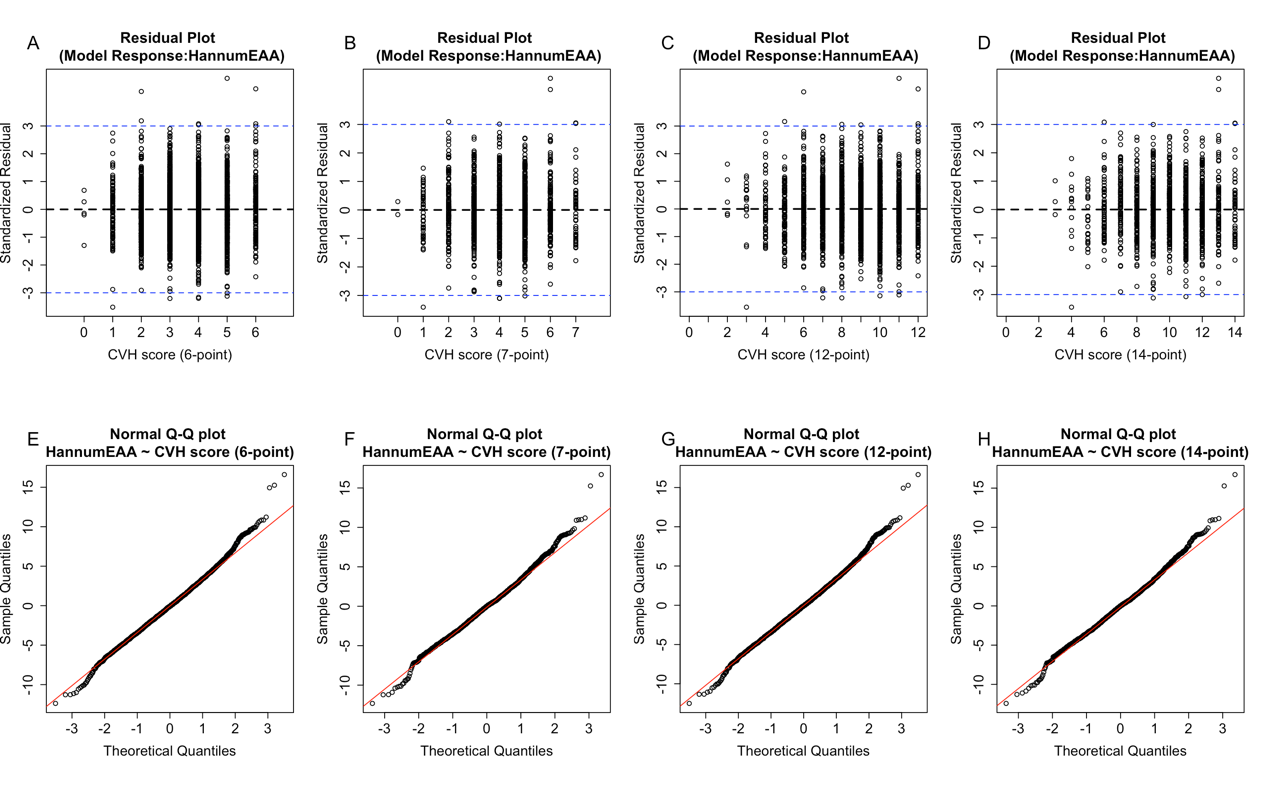
**

**Figure S3. Residual and Normal Quantile-Quantile plots for regression models based on PhenoEAA.** (A-D) Residual plots for regressing PhenoEAA on: (A) 6-point CVH score; (B) 7-point CVH score; (C) 12-point CVH score; (D) 14-point CVH score. (E-H) The Normal Q-Q plots for models regressing PhenoEAA on: (E) 6-point CVH score; (F) 7-point CVH score; (G) 12-point CVH score; (H) 14-point CVH score. Sex, drinking status, and educational attainment have been adjusted in all models. No substantial violation of the assumption of normality or constant variance was observed for any model.


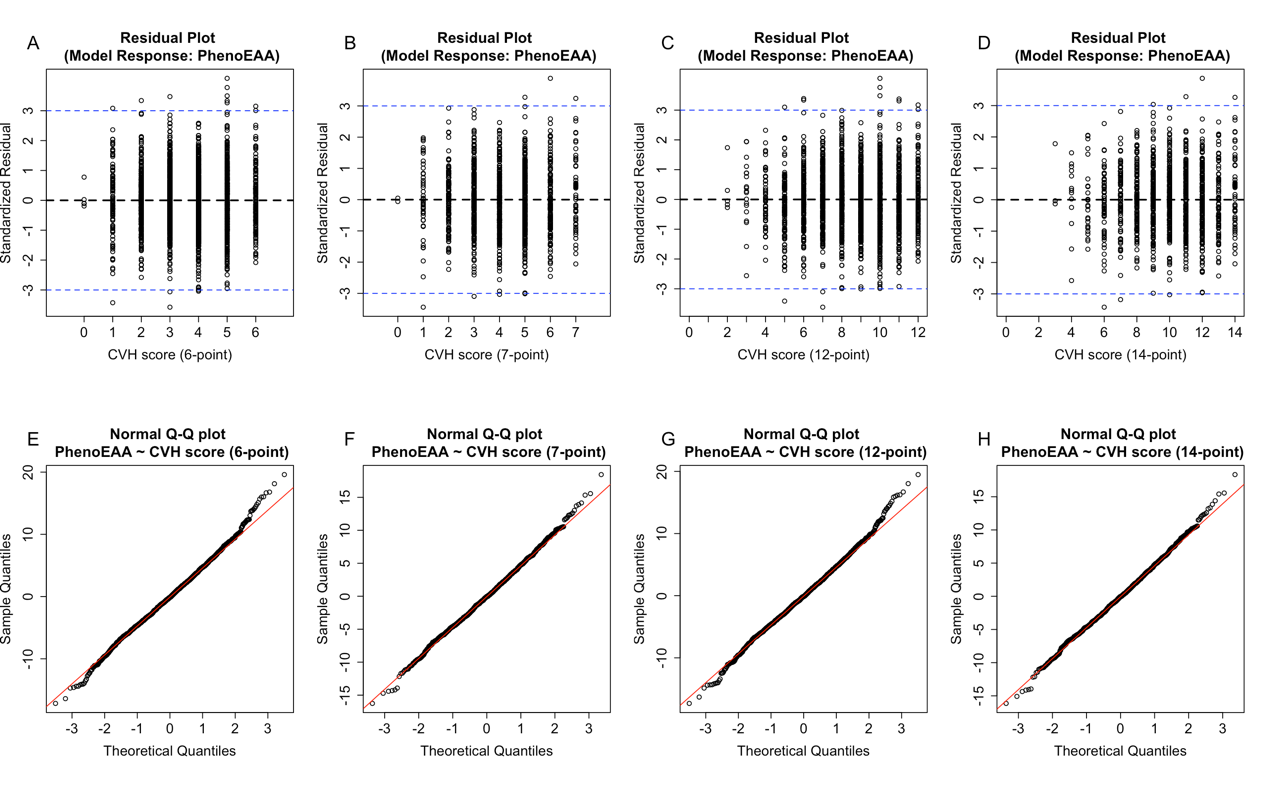


**Figure S4. Residual and Normal Quantile-Quantile plots for regression models based on GrimEAA.** (A-D) Residual plots for regressing GrimEAA on: (A) 6-point CVH score; (B) 7-point CVH score; (C) 12-point CVH score; (D) 14-point CVH score. (E-H) The Normal Q-Q plots for models regressing GrimEAA on: (E) 6-point CVH score; (F) 7-point CVH score; (G) 12-point CVH score; (H) 14-point CVH score. Sex, drinking status, and educational attainment have been adjusted in all models. No substantial violation of the assumption of constant variance was observed for any model. However, the QQ plots showed that the residuals followed distributions with heavier tails than the normal distribution.


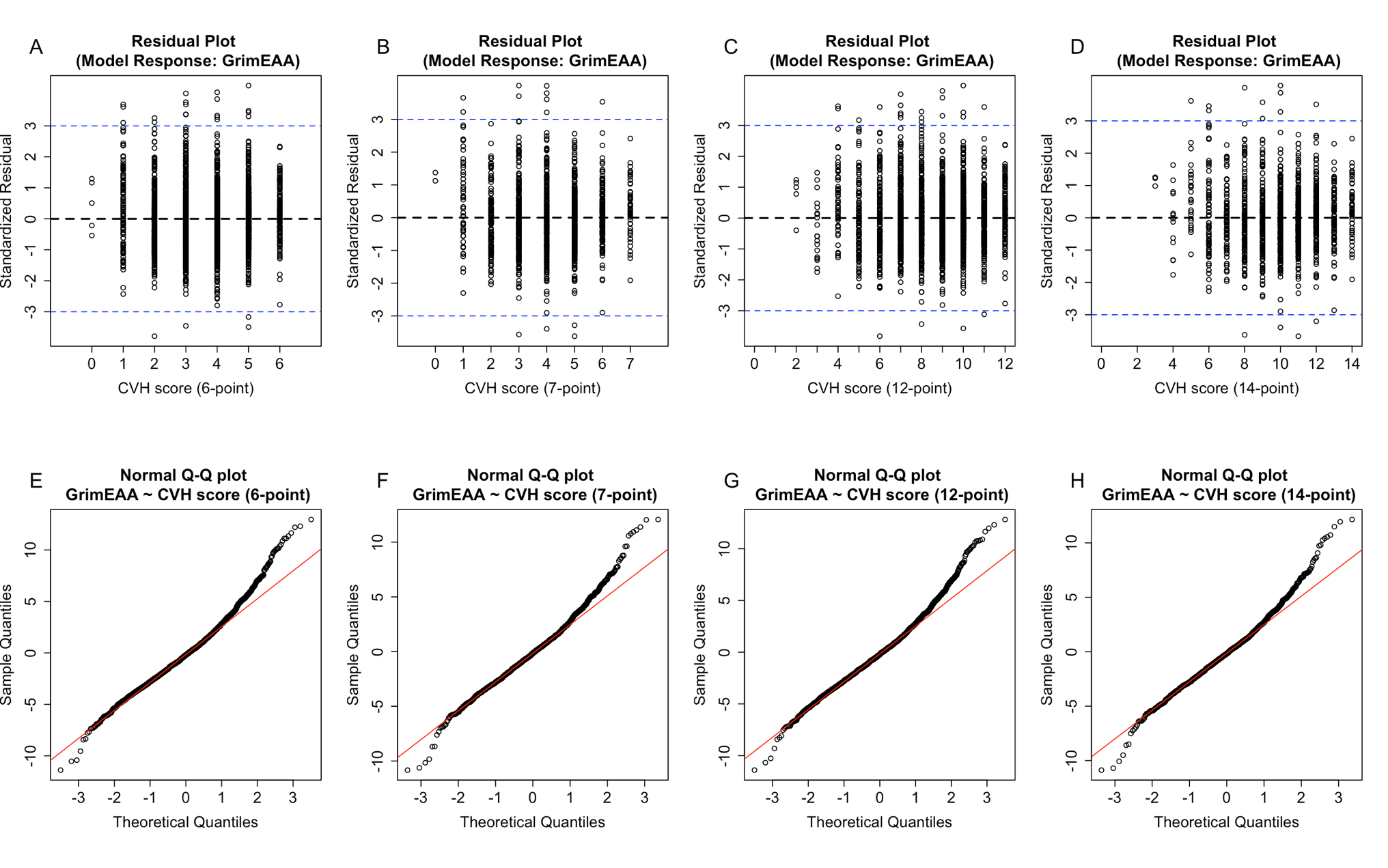


**Figure S5. Residual and Normal Quantile-Quantile plots for rank-based inverse normal transformation (rank-based INT) of IEAA.** (A-D) Residual plots for regressing rank-based INT of IEAA on: (A) 6-point CVH score; (B) 7-point CVH score; (C) 12-point CVH score; (D) 14-point CVH score. (E-H) The Normal Q-Q plots for models regressing rank-based INT of IEAA on: (E) 6-point CVH score; (F) 7-point CVH score; (G) 12-point CVH score; (H) 14-point CVH score. Sex, drinking status, and educational attainment have been adjusted in all models. No substantial violation of the assumption of normality or constant variance was observed for any model.


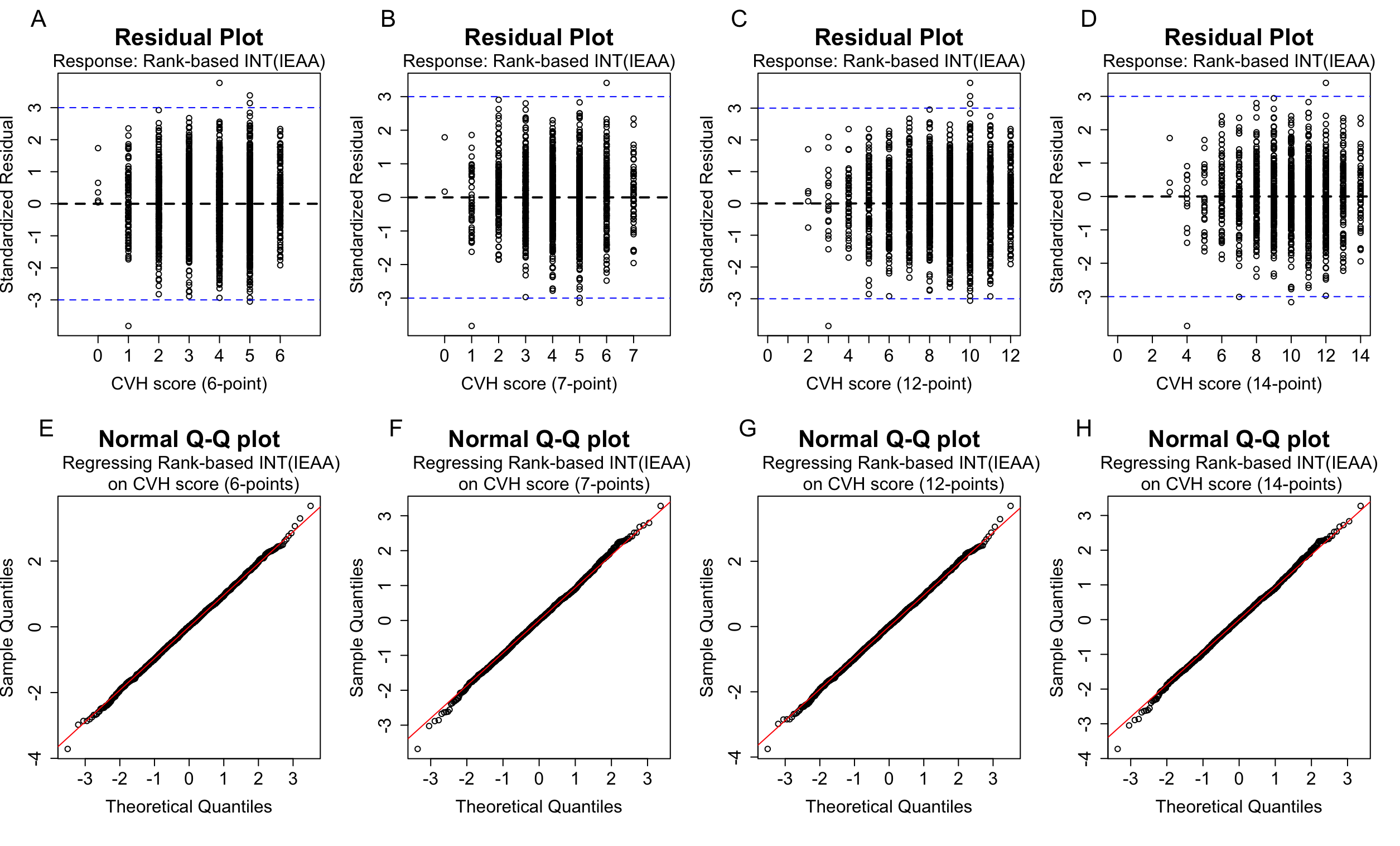


**Figure S6. Residual and Normal Quantile-Quantile plots for rank-based inverse normal transformation (rank-based INT) of HannumEAA.** (A-D) Residual plots for regressing rank-based INT of HannumEAA on: (A) 6-point CVH score; (B) 7-point CVH score; (C) 12-point CVH score; (D) 14-point CVH score. (E-H) The Normal Q-Q plots for models regressing rank-based INT of HannumEAA on: (E) 6-point CVH score; (F) 7-point CVH score; (G) 12-point CVH score; (H) 14-point CVH score. Sex, drinking status, and educational attainment have been adjusted in all models. No substantial violation of the assumption of normality or constant variance was observed for any model.


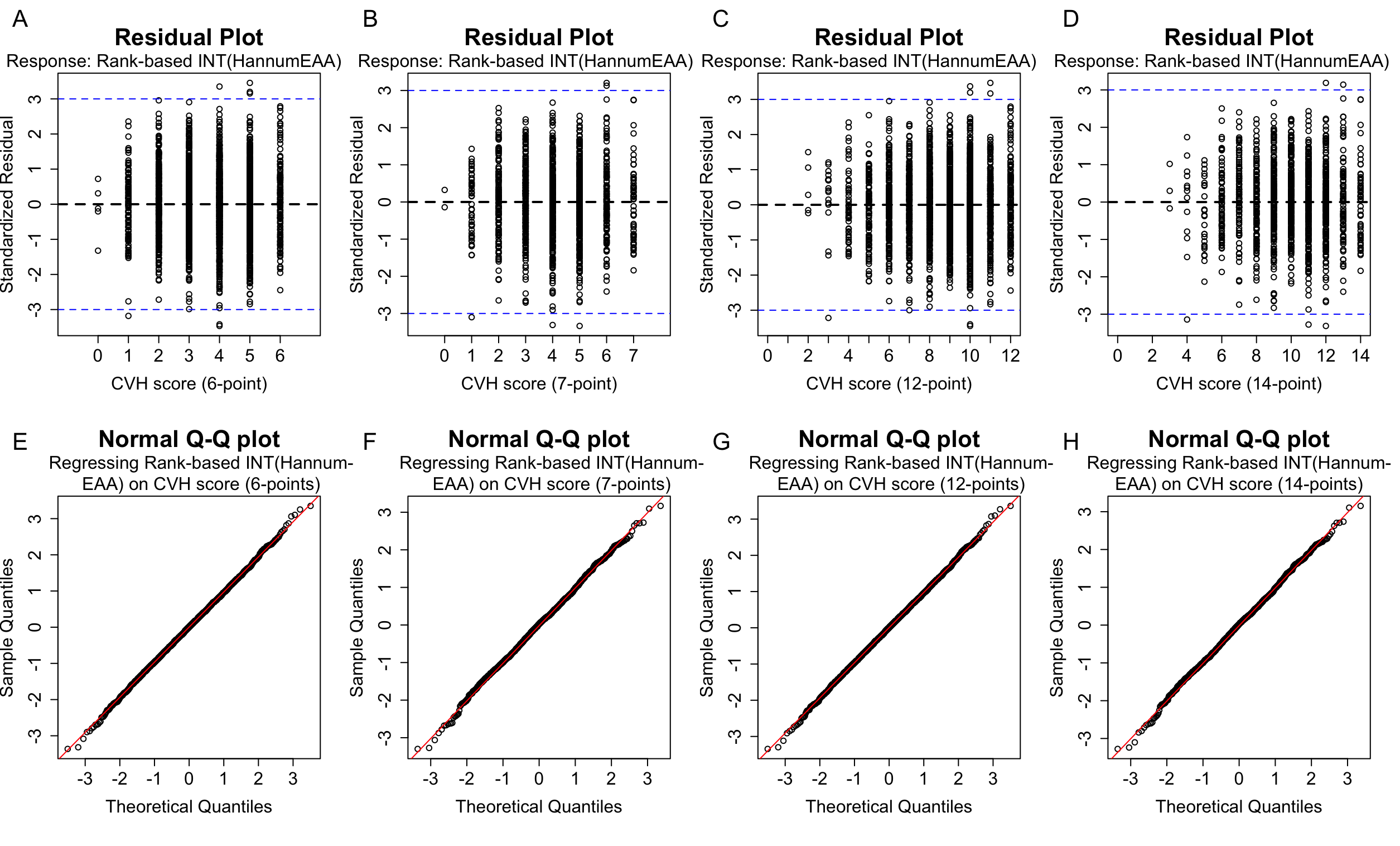


**Figure S7. Residual and Normal Quantile-Quantile plots for rank-based inverse normal transformation (rank-based INT) of PhenoEAA.** (A-D) Residual plots for regressing rank-based INT of PhenoEAA on: (A) 6-point CVH score; (B) 7-point CVH score; (C) 12-point CVH score; (D) 14-point CVH score. (E-H) The Normal Q-Q plots for models regressing rank-based INT of PhenoEAA on: (E) 6-point CVH score; (F) 7-point CVH score; (G) 12-point CVH score; (H) 14-point CVH score. Sex, drinking status, and educational attainment have been adjusted in all models. No substantial violation of the assumption of normality or constant variance was observed for any model.


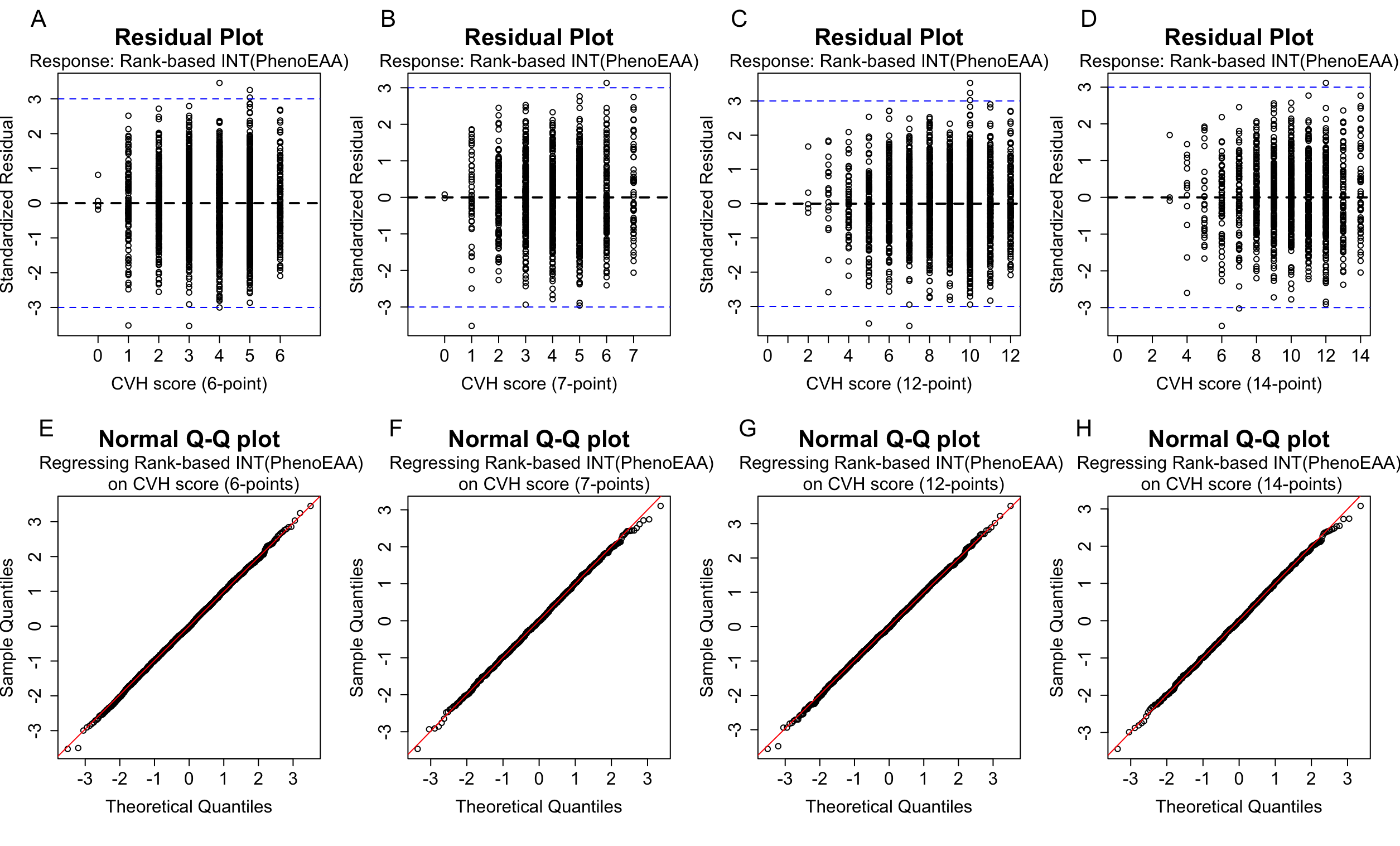


**Figure S8. Residual and Normal Quantile-Quantile plots for rank-based inverse normal transformation (rank-based INT) of GrimEAA.** (A-D) Residual plots for regressing rank-based INT of GrimEAA on: (A) 6-point CVH score; (B) 7-point CVH score; (C) 12-point CVH score; (D) 14-point CVH score. (E-H) The Normal Q-Q plots for models regressing rank-based INT of GrimEAA on: (E) 6-point CVH score; (F) 7-point CVH score; (G) 12-point CVH score; (H) 14-point CVH score. Sex, drinking status, and educational attainment have been adjusted in all models. No substantial violation of the assumption of normality or constant variance was observed for any model.


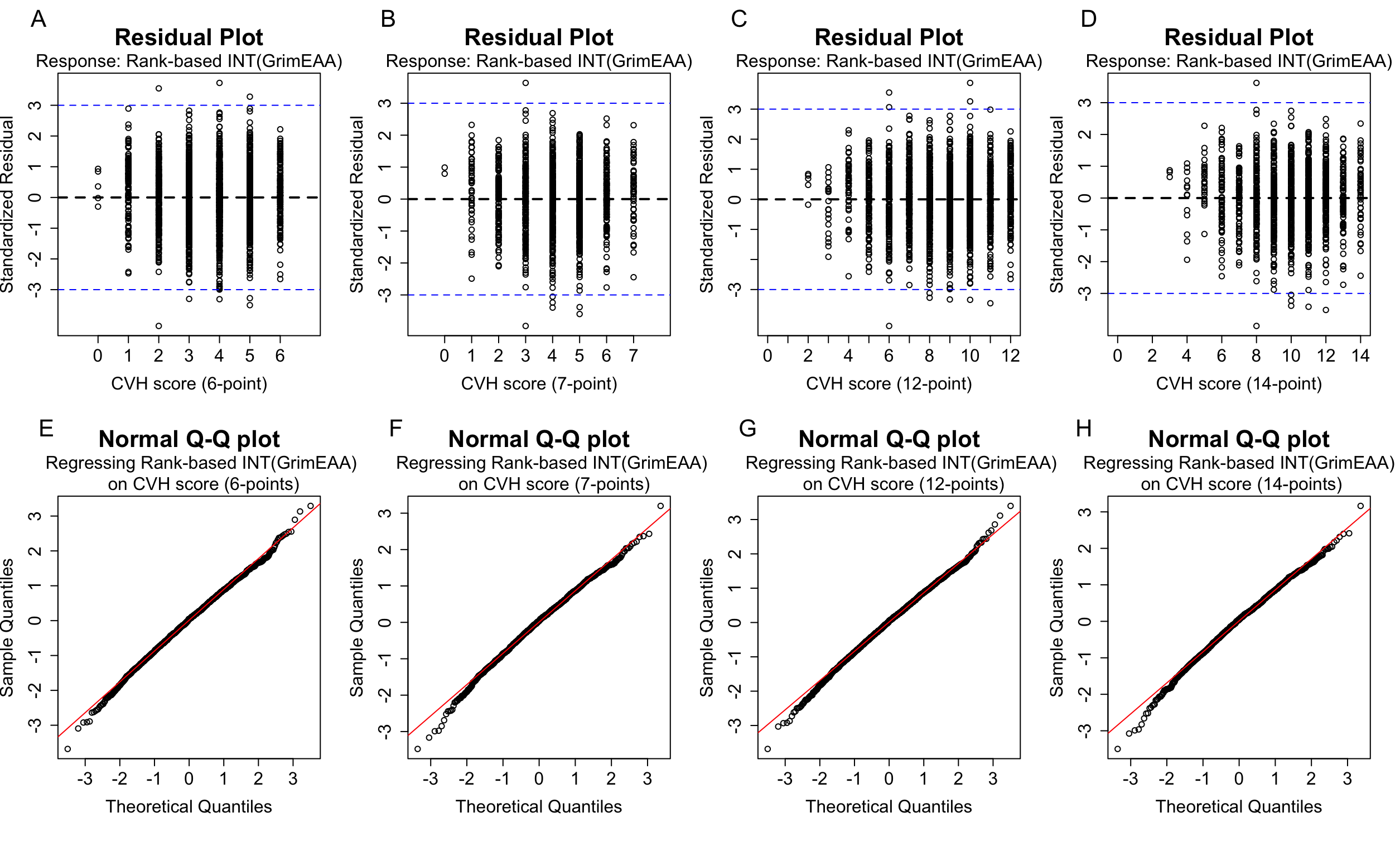

Supplement: Supplementary file 1 — Additional file 1. Table S1. Basic characteristics of the 2474 TWB participants stratified by tertiles of PhenoEAA. Table S2. Basic characteristics of the 2474 TWB participants stratified by tertiles of IEAA. Table S3. Basic characteristics of the 2474 TWB participants stratified by tertiles of HannumEAA. Table S4. The 17 diet-related questions in the TWB questionnaire. Table S5. Regression coefficients of all the covariates included in statistical models. Table S6. Variance inflation factors (VIF) to check multicollinearity. Table S7. Regressing rank-based inverse normal transformation of the four measures of EAA on the CVH score. Figure S1. Residual and Normal Quantile-Quantile plots for regression models based on IEAA. Figure S2. Residual and Normal Quantile-Quantile plots for regression models based on HannumEAA. Figure S3. Residual and Normal Quantile-Quantile plots for regression models based on PhenoEAA. Figure S4. Residual and Normal Quantile-Quantile plots for regression models based on GrimEAA. Figure S5. Residual and Normal Quantile-Quantile plots for rank-based inverse normal transformation (rank-based INT) of IEAA. Figure S6. Residual and Normal Quantile-Quantile plots for rank-based inverse normal transformation (rank-based INT) of HannumEAA. Figure S7. Residual and Normal Quantile-Quantile plots for rank-based inverse normal transformation (rank-based INT) of PhenoEAA. Figure S8. Residual and Normal Quantile-Quantile plots for rank-based inverse normal transformation (rank-based INT) of GrimEAA. [file 13148_2022_1295_MOESM1_ESM.docx]
